# Insect frass fertilizer as a regenerative input for improved biological nitrogen fixation and sustainable bush bean production

**DOI:** 10.3389/fpls.2024.1460599

**Published:** 2024-09-05

**Authors:** Agnes Chepkorir, Dennis Beesigamukama, Harun I. Gitari, Shaphan Y. Chia, Sevgan Subramanian, Sunday Ekesi, Birachi Eliud Abucheli, Jean Claude Rubyogo, Theodore Zahariadis, Gina Athanasiou, Aikaterini Zachariadi, Vasileios Zachariadis, Abdou Tenkouano, Chrysantus M. Tanga

**Affiliations:** ^1^ International Centre of Insect Physiology and Ecology, Nairobi, Kenya; ^2^ Department of Agricultural Science and Technology, Kenyatta University, Nairobi, Kenya; ^3^ Department of Zoology and Entomology, University of Pretoria, Pretoria, South Africa; ^4^ International Centre for Tropical Agriculture, Kigali, Rwanda; ^5^ International Centre for Tropical Agriculture, Nairobi, Kenya; ^6^ Synelixis Solutions S.A., Chalkida, Greece; ^7^ Department of Agriculture Development, National and Kapodistrian University of Athens, Athens, Greece

**Keywords:** BSF frass fertilizer, symbiotic nitrogen fixation, bush bean yield, circular economy, soil health, climate-smart fertilizer

## Abstract

Bush bean (*Phaseolus vulgaris* L.) production is undermined by soil degradation and low biological nitrogen fixation (BNF) capacity. This study evaluated the effect of black soldier fly frass fertilizer (BSFFF) on bush bean growth, yield, nutrient uptake, BNF, and profitability, in comparison with commercial organic fertilizer (Phymyx, Phytomedia International Ltd., Kiambu, Kenya), synthetic fertilizer (NPK), and rhizobia inoculant (Biofix, MEA Fertilizers, Nairobi, Kenya). The organic fertilizers were applied at rates of 0, 15, 30, and 45 kg N ha^−1^ while the NPK was applied at 40 kg N ha^−1^, 46 kg P ha^−1^, and 60 kg K ha^−1^. The fertilizers were applied singly and in combination with rhizobia inoculant to determine the interactive effects on bush bean production. Results showed that beans grown using BSFFF were the tallest, with the broadest leaves, and the highest chlorophyll content. Plots treated with 45 kg N ha^−1^ BSFFF produced beans with more flowers (7 – 8%), pods (4 – 9%), and seeds (9 – 11%) compared to Phymyx and NPK treatments. The same treatment also produced beans with 6, 8, and 18% higher 100-seed weight, compared to NPK, Phymyx, and control treatments, respectively. Beans grown in soil amended with 30 kg N ha^−1^ of BSFFF had 3–14-fold higher effective root nodules, fixed 48%, 31%, and 91% more N compared to Phymyx, NPK, and rhizobia, respectively, and boosted N uptake (19 – 39%) compared to Phymyx and NPK treatments. Application of 45 kg N ha^−1^ of BSFFF increased bean seed yield by 43%, 72%, and 67% compared to the control, NPK and equivalent rate of Phymyx, respectively. The net income and gross margin achieved using BSFFF treatments were 73 – 239% and 118 – 184% higher than the values obtained under Phymyx treatments. Our findings demonstrate the high efficacy of BSFFF as a novel soil input and sustainable alternative for boosting BNF and improving bush bean productivity.

## 1 Introduction

Globally, bush beans (*Phaseolus vulgaris* L.) are highly-valued pulse crops for their critical role in food and nutrition security, income generation, and soil health management (Broughton et al., 2003). Its dietary roles as human food (Araujo et al., 2020) and livestock feed (Ojiewo et al., 2015) make it a salient legume in addressing food insecurity (Katungi et al., 2010). The health risks associated with the consumption of animal proteins have prompted a shift to safer legume-based proteins such as bush beans (Daryanto et al., 2015). Most small-scale farmers incorporate bush beans in cropping systems to boost and maintain soil fertility through biological nitrogen (N) fixation, soil moisture conservation, and erosion control (Sousa et al., 2022).

Bush bean yield remains low partly due to soil nutrient depletion, high fertilizer costs, biotic stress factors (pests and diseases), and climate change effects (low and unreliable rainfall and increased temperatures) (Wanjala et al., 2019; Beebe et al., 2013). The situation has been worsened by the alarming levels of soil degradation coupled with skyrocketing fertilizer prices (FAO, IFAD, UNICEF, WFP and WHO, 2022). For optimal growth and yield, bush beans require 40 kg N ha^-1^ (Chekanai et al., 2018), 46 kg phosphorus (P) ha^-1^ (Koskey et al., 2017), 60 kg potassium (K) ha^-1^ (Tsige et al., 2022), and adequate micronutrients per season. However, the demand cannot be met due to the highly degraded nature of sub-Saharan Africa (SSA) soil. Estimates show that around 29% of arable land in sub–Saharan Africa has undergone intense degradation (Zingore et al., 2015; Nungula et al., 2023) due to poor farming activities. Soils in sub–Saharan Africa lose 10 – 70 kg of N, 2 – 10 kg of P, and 8 – 50 kg of K (Stoorvogel and Smaling, 1998; Nyawade et al., 2019), which is a major setback to agricultural production (Mohammed et al., 2020; Chirwa et al., 2015). To replenish these nutrients, farmers should use organic and mineral fertilizers, as well as bio-fertilizers. However, available organic fertilizers have poor quality (Ndambi et al., 2019; Jensen et al., 2012), while mineral fertilizers are costly, inaccessible, and require a lot of energy to produce them to supply the secondary and micronutrients required for optimal crop production (Fairhurst, 2012). Additionally, the use of mineral fertilizers has raised a lot of concerns due to their negative impact on human health and the environment (Mutuma et al., 2014; Sairaam et al., 2023).

Rhizobia bio-fertilizers naturally supply the N required by beans through the process of biological N fixation (BNF). However, BNF is less effective in most soils due to low populations of effective rhizobia strains, arising from gross reduction in soil biodiversity. The effectiveness of most commercial rhizobia inoculants is affected by competition from the indigenous rhizobacteria (Araujo et al., 2020), soil acidity, moisture stress, high temperatures, and deficiency of the nutrients required for a successful BNF process (Zaheer et al., 2022; Hungria et al., 2013). Eventually, farmers do not benefit from the application of rhizobia-based biofertilizers and continue to experience decreased bush bean yields (Kyomuhendo et al., 2018). Phosphorus is the most critical nutrient required for BNF (Stevens et al., 2019) due to its significant role in supplying the energy required during the process (Schulze et al., 2006). Most tropical soils are acidic and deficient in P (Montanarella et al., 2016; Mugo et al., 2021), therefore not readily available for the plant. Also, acidic soils suppress nodule formation, survival of rhizobium bacteria (Wang et al., 2022; Mirriam et al., 2023), and their infection of the root nodules to cause N fixation (Morón et al., 2005).

Organically managed soils have been reported to boost the BNF process through increased populations, biomass, and activities of free-living N-fixing bacteria (Wessén et al., 2010), majorly due to abundant organic carbon (C) levels that act as energy sources (Fernández-Luqueño et al., 2010). The BNF process can supply around 700 kg of N per hectare in legume cropping systems (Soumare et al., 2020; Herridge et al., 2008). Symbiosis alone can contribute 80% of N fixed on croplands (Mendoza-Suárez et al., 2020; Herridge et al., 2008). This demonstrates the sustainability and economic viability associated with the utilization of N fixation in place of mineral fertilizers for legume crop production (Araujo et al., 2020; Carranca, 2017; Jena et al., 2022). Succeeding crops can also benefit from the fixed nitrogen (Mirriam et al., 2022; Chapagain and Riseman, 2014), due to the high N transfer rate of up to 85% (Paynel et al., 2008; Moyer-Henry et al., 2006). This reduces the requirement of using costly fertilizers which are beyond reach by most smallholder farmers.

Novel and high-quality organic fertilizers such as insect frass fertilizer, which is derived from valorization of organic wastes using saprophytic insect larvae as bioconverters can restore soil health, boost BNF, and increase bush bean yield. Insect frass fertilizer is rich in nutrients, and beneficial microbes, besides being pathogen-free and requiring a short production time (Beesigamukama et al., 2022; Beesigamukama et al., 2021a; Adin Yéton et al., 2019; Lalander et al., 2015). Past studies have demonstrated the potential of insect frass fertilizer to boost beneficial soil microbes, suppress pests and diseases (Tanga and Kababu, 2023; Kemboi et al., 2022), enhance water-holding capacity, soil aeration, and soil aggregation (Beesigamukama et al., 2020b; Pagliai et al., 2004), reduce soil acidity and increase availability of nutrients, especially P, which is key for BNF (Anyega et al., 2021; Beesigamukama et al., 2021b). Additionally, frass fertilizer application suppresses soil-borne fungal pathogens such as *Fusarium, Pythium*, and *Rhizoctonia*, in different crops, including bush beans (Kemboi et al., 2022; Quilliam et al., 2020).

Application of BSFFF improves soil microbial activities better than commercial fertilizers (Gebremikael et al., 2015; Kagata and Ohgushi, 2012) and boosts soil ammonium (Beesigamukama et al., 2021b), which is highly preferred by microorganisms as an energy source. Previous studies have also demonstrated that BSFFF enhances plant tolerance to moisture stress (Abiya et al., 2022) and boosts flower formation and pollination (Barragán-Fonseca et al., 2022). The enhanced soil health benefits have resulted in higher nutrient uptake, yield, and economic returns from crops grown using black soldier fly frass fertilizer, compared to mineral fertilizers (Beesigamukama et al., 2022; Agustiyani et al., 2021; Anyega et al., 2021; Tanga et al., 2022).

Although BSFFF has demonstrated high potential in enhancing the growth of cereals and vegetable crops (Anyega et al., 2021; Beesigamukama et al., 2020a; Quilliam et al., 2020; Choi et al., 2009), there is limited evidence about the agronomic potential of this organic fertilizer on leguminous crops such as bush beans. The few studies involving BSFFF and legumes focused on French beans and did not determine BNF and profitability (Anyega et al., 2021; Quilliam et al., 2020). In as much as soil N is required, its overuse suppresses BNF through poor nodulation and inhibition of key enzyme activities especially nitrogenase (Reinprecht et al., 2020; Mingotte et al., 2019). As such, the optimal application rate of BSFFF for high bush bean yield, BNF, and profit margins remains largely unknown. There is limited research attention on the effect of the combined application of BSFFF and commercial rhizobia inoculant on BNF and bean yield, and the optimal BSFFF rate that can supply the starter N dose required during the BNF process. Such information would guide policy makers, researchers, non-government organizations, the private sector, and farmers in the promotion and effective use of BSFFF as a regenerative fertilizer input for improved bush bean production. Therefore, this study was undertaken to determine the effect of BSFFF on bush bean growth, yield, nitrogen uptake, BNF, and profitability, in comparison with commercial organic fertilizer, mineral fertilizer, and rhizobia-based biofertilizer.

## 2 Materials and methods

### 2.1 Experimental site

Field studies were conducted for two consecutive seasons (October – December 2022 and March – June 2023) at Kenyatta University Teaching and Demonstration Farm (36°55′34″ N, and 1°10′59″ E, 1720 meters above sea level), Kiambu County, Kenya. The area receives bimodal rainfall; the short rain season runs from October to December, while long rains are received between March and June. The annual average rainfall is 850 mm. Kiambu receives mean monthly temperature and relative humidity of 20 – 27°C and 65 – 84%, respectively. The area is relatively warm and is characterized by Ferralsols (FAO-UNESCO, 1990), with shallow depths, low organic matter content, and medium acidity. Land use history indicated that the site is used for annual crop production where sorghum was the previous crop.

Before experiments, soil sampling was conducted at a depth of 0–30 cm using a zig-zag method and soil auger. The field was subdivided into three sections and ten cores were collected per section; quarter sampling was used to collect a composite sample per section. The samples were air-dried for 5 days, and ground using mortar and pestle, pending analysis of selected physical and chemical properties using standard methods. Soil texture was determined using the Bouyoucos method following procedures described by Okalebo et al. (2002). Available P and total nitrogen were determined using Bray 1 method and using Kjedahl digestion and distillation method, respectively. Soil pH was measured using soil to water suspension (2.5:1), and pH and EC values were read directly using pH (AD1000, Adwa, Bucharest, Romania) and EC meter (AVI, Labtech, Mumbai, India), following procedures of Okalebo et al. (2002). Soil organic carbon (SOC) was determined following the Walkley-Black (wet oxidation) method (Walkley and Black, 1934). As per Jackson and Mahmood (1994), exchangeable cations (K, magnesium [Mg], sodium [Na], and calcium [Ca]) were determined using an Atomic Absorption Spectrophotometer (AAS). **Table 1** presents the selected characteristics of the soil used in the study.

**Table 1:** Selected chemical and physical characteristics of soil and organic fertilizers used during the field study.

Parameter	Test soil	Black soldier fly frass fertilizer	Phymyx
pH	6.05	9.62	6.62
EC (mS cm^-1^)	28.27	25.47	5.89
Total nitrogen (g kg^-1^)	1.01	19.78	15.5
Total organic carbon (g kg^-1^)	13.3	334.7	167
Organic matter (g kg^-1^)	21	893	648
Phosphorus (mg kg^-1^)	7.09	12.2	10.1
Potassium (mg kg^-1^)	468.3	43.47	16.2
Magnesium (mg kg^-1^)	271.3	6.63	4.2
Iron (mg kg^-1^)	207.33	12167	20400
Copper (mg kg^-1^)	0.73	31.23	21.7
Zinc (mg kg^-1^)	4.09	189.67	231
Boron (g kg^-1^)	0.24	46.5	39.4
C/N ratio	–	11.7	10.8
Ammonium (g kg^-1^)	–	–	–
Nitrate (g kg^-1^)	–	–	–
Sand (%)	58.2	–	–
Clay (%)	25.8	–	–
Silt (%)	16	–	–
Textural class	Sandy clay loam		

**Figure 1:**
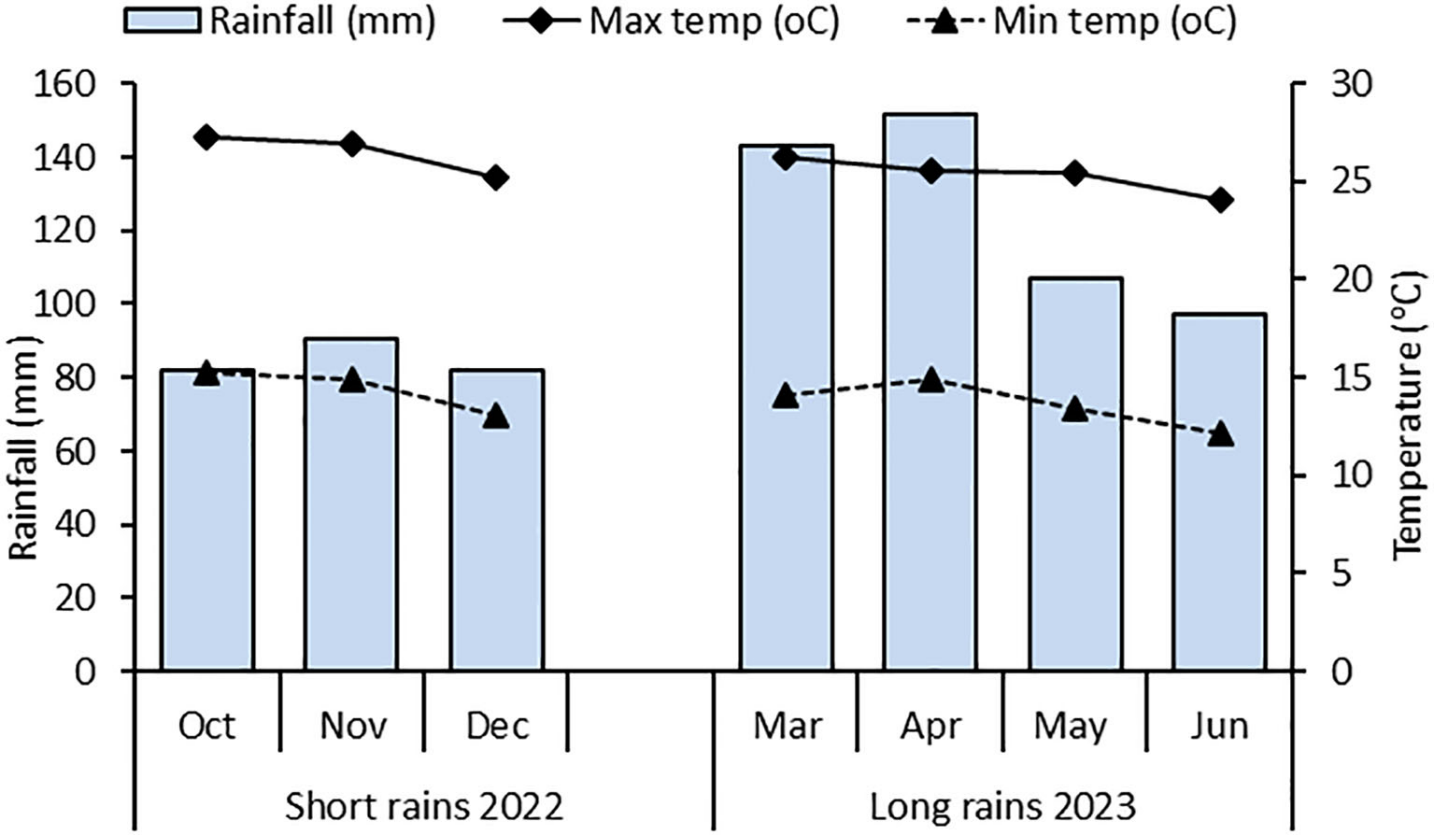
Monthly average temperature (Minimum and maximum) and total monthly rainfall during field experiments.

Throughout the study period, the total monthly rainfall and average monthly temperature data were collected from Kenyatta University weather station. The mean monthly temperatures of 25.2– 27.3°C and 24.1 – 26.2°C were recorded during the short and long rain seasons, respectively. The total amount of rainfall received was 243 mm, and 499 mm during the short and long rain seasons, respectively (**Figure 1**). Rainfall intensity was highest in November and April for the 2022 short rains and 2023 long rains, respectively.

### 2.2 Source of experimental materials

The experiment consisted of four fertilizers: Black soldier fly frass fertilizer (BSFFF), commercial organic fertilizer (Phymyx) (Phytomedia International Limited, Kiambu County, Kenya), biofertilizer (Rhizobia inoculant) (Biofix) (MEA Fertilizers, Nairobi, Kenya) and mineral fertilizer (NPK). The BSFFF was produced by composting frass from the black soldier fly (BSF) colony maintained in the animal rearing and quarantine unit of the International Center of Insect Physiology and Ecology (*icipe*). The BSF frass was obtained by feeding BSF larvae on potato waste following procedures by Beesigamukama et al. (2020) and Shumo et al. (2019). The frass was composted for five weeks using the heap method, resulting in a mature and stable organic fertilizer product (Beesigamukama et al., 2021a). The organic fertilizers were subjected to laboratory analysis using standard procedures and results are presented in **Table 1**. Bush bean seeds (Rosecoco GLP2 bush bean seeds) were procured from Simlaw Seed Company Limited, Nairobi. This is a high-yielding variety recommended for medium and low-altitude areas of Kenya (KALRO, 2008). The NPK fertilizers (urea, DAP, MOP) were sourced from Kenya Farmers Association (KFA) stores in Nairobi.

### 2.3 Treatments and experimental setup

There were 15 treatments, grouped into two sets. In the first set of treatments, BSFFF and Phymyx were applied at three rates equivalent to 15, 30, and 45 kg N ha^−1^ to determine the optimal N application rate for improved bush bean production. The rates were denoted as 15BSFFF, 30BSFFF, and 45BSFFF for 15, 30, and 45 kg N ha^−1^ BSFFF treatments, respectively, and the respective rates for Phymyx were indicated as 15PHY, 30PHY, and 45PHY. The rates of 15, 30, and 45 kg N ha^−1^ were supplied by applying 522.6, 1045.3, and 1567.9 kg ha^−1^ of BSFFF, respectively, and 967.7, 1935.5, and 2903.2 kg ha^−1^ of Phymyx, respectively. The mineral fertilizer treatment applied at rates of 40 kg N ha^−1^, 46 kg P ha^−1^, and 60 kg K ha^−1^ (Chekanai et al., 2018) was included for comparison purposes. The N, P, and K were supplied using urea (47.8 kg ha^−1^), diammonium phosphate (100 kg ha^−1^), and muriate of potash (120.5 kg ha^−1^), respectively. In the second set of treatments, the mineral and organic fertilizers were applied at the rates described above and combined with rhizobia (Rh) inoculant to determine their interaction effects on bush bean growth, yield, and BNF. These treatments were denoted as Rh+15BSFFF, Rh+30BSFFF, and Rh+45BSFFF for BSFFF, Rh+15PHY, Rh+30PHY, Rh+45PHY for Phymyx, and Rh+NPK for mineral fertilizer. The rhizobia biofertilizer was applied by inoculating bean seeds following the manufacturer’s instructions. The seeds requiring inoculation were first coated with 5% gum Arabic to make them sticky, and later mixed with the inoculum at a rate of 100 g of inoculum per 15 kg of bean seeds (Maingi et al., 2001). The control treatment consisted of unfertilized soil and uninoculated seeds.

The experiment was laid out using a randomized complete block design (RCBD) and replicated three times. Plot sizes of 3 m × 3 m with border width of 0.5 m and inter-block distance of 1 m were used. The bush bean variety Rosecoco GLP2 was used as the test crop. The beans were planted in rows spaced 0.5 m apart with two seeds being sowed per hill at an inter-hill spacing of 0.1 m (Gicharu et al., 2013). The organic and mineral fertilizers were applied using the band method; the organics were applied a day before planting, while the mineral fertilizers were applied at planting. Gap filling was conducted 7 days after planting to maintain a uniform plant population across treatments. Across all the treatments, weeding was conducted using a hand hoe, whereas pests such as aphids, and blight disease were controlled by spraying the crop with recommended pesticides (Prove 1.9 EC) and fungicides (Funguran-OH 50WP) at vegetative stage. The pesticide was diluted at a rate of 15ml per20 litres of water, while the fungicide was dissolved at a rate of 50 grams per 20 litres of water.

### 2.4 Data collection

#### 2.4.1 Bean growth

Data was collected on leaf length, number of leaves, chlorophyll content, plant height, leaf width, and stem diameter using 10 plants that were randomly selected from the four inner rows of each plot. Data was collected at the following phenological stages: early vegetative growth (14 days after planting) (dap), flower formation (28 dap), pod formation (42 dap), seed filling (56 dap), and senescence (70 dap). Chlorophyll content was measured using a Soil and Plant Analysis Development (SPAD) meter (Konica-Minota 502, Chiyoda, Japan) placed on three of the topmost fully grown leaves. Plant height was measured using a tape measure from the soil surface to the plant tip. The number of leaves was determined by counting the photosynthetically active leaves. Leaf width was measured using a tape measure placed at the broadest part of the leaf whereas leaf length was measured from the petiole base to apex. Leaf areas were the product of leaf width and leaf length. Stem girth was measured using a digital vernier caliper (Silverline Digital Vernier Caliper 300 mm, Toolstream Ltd, Yeovil, UK) placed on the plant stem at 5 cm from the soil surface. The number of flowers was determined at 42 dap by hand counting while pod length number of pods per plant, and that of seeds per pod were determined at the physiological maturity stage (56 dap).

#### 2.4.2 Bean yield

Yield data was collected at the harvesting stage (79 dap) when the pods were completely dry. At harvesting, plants were carefully uprooted to avert seeds from scattering. Shelling was done manually, and fresh weights of the seeds and haulms per plot were measured using a weighing scale. Sub samples of 500 g each for seeds and haulms were air-dried for 72 hours and oven-dried at 60°C for 48 hours to drive off all the moisture. The dry weights obtained were used to determine moisture content and dry matter yield on a hectare basis (t ha^−1^). Furthermore, 100 randomly selected seeds per treatment were weighed to determine the 100 seed weight (Khanam and Platel, 2016).

#### 2.4.3 Nutrient uptake

Representative samples of the bean seeds and haulms were collected randomly, air-dried for 72 hours and oven-dried at 60°C for 48 hours to a constant weight. The dried samples were ground into powder using an analytical mill and subjected to laboratory analysis to determine the concentrations of N, P, and K using standard laboratory procedures (Okalebo et al., 2002). The nutrient concentrations and yield data obtained in section 2.3 were used to determine nutrient uptake in seeds (Equation 1) and haulms (Equation 2). Total nutrient uptake was computed as the sum of uptake in seeds and haulms.


(1) $$\text{Nutrient uptake in seeds}\;\left(kg\;ha^{-1}\right)=\frac{\text{nutrient concentration }\left(\%\right)\;\times \text{ seed yield }({\text{Kg ha}}^{-1})}{100}\;$$

(2) $$\text{Nutrient uptake in haulms }\left(kg\;ha^{-1}\right)=\frac{\text{nutrient concentration in haulms }\left(\%\right)\;\times \text{ halum yield }({\text{Kg ha}}^{-1})}{100}\;$$

#### 2.4.4 Determination of biological nitrogen fixation

Biological N fixation was assessed by determining the total number of nodules, number of effective nodules, nodule weight per plant, and quantity of N fixed. The total and effective root nodules were assessed between 50% flowering and early pod formation (42 – 56 dap), this is the stage when active nodule formation occurs (Zhou et al., 2021). Ten randomly selected plants per plot were carefully uprooted to avoid nodule detachment. The total number of root nodules per plant was determined using a tally counter. The nodules were cautiously detached from the roots and placed in plastic sampling bags that were tightly sealed, transported to the laboratory in a cooler box containing dry ice, and stored at -20°C (Korir et al., 2017) to keep them fresh pending nodule assessment. Physical examination, which involved slicing the nodules using a sterile scalpel was done. Any sliced nodule that had pink pigmentation (leghaemoglobin) was considered effective, while those that had other colorations were characterized as ineffective (Jourand et al., 2004).

The amount of N fixed by bush beans was determined using the N-difference method, which computes the difference between the total N uptake of the N-fixing crop (legume) and the non-N-fixing crop (Peoples et al., 1989). Maize (Katumani variety) was used as the reference crop (Cafaro La Menza et al., 2020) since maize (*Zea mays* L.) and beans have similarities in root architecture (Ordóñez et al., 2018; Nichols et al., 2019). Also, the maturity period of the maize variety used (2.5 – 3 months) is similar to that of the bush bean variety used in this study. Maize was planted in unfertilized plots at a spacing of 75 cm between rows and 30 cm between plants. At maturity, maize yield, and N uptake in grain and stover were determined using standard procedures. The quantity of N fixed by beans was determined using Equation 3.


(3) $$\text{Nitrogen fixed }\left(\text{kg }ha^{-1}\right)={\text{TNC}}_{\text{fc}}\;-{\text{TNC}}_{\text{nfc}}$$

Where TNC_fc_ and TNC_nfc_ represent the total N uptake of the fixing (beans) and non-fixing (maize) crops, respectively.

#### 2.4.5 Economic returns to bush bean production

The profitability of bush bean production using BSFFF, and commercial fertilizer was assessed by determining net income, benefit to cost ratio, return on investment, and gross margins. Net income was determined as the difference between variable costs and revenue. The variable costs considered during the study included the cost of seeds, fertilizers, rhizobia inoculants, labor, pesticides, and fungicides). The average prices of fertilizers and seeds were sourced from the Kenya Farmers’ Association and Africa Fertilizer Website (Africa Fertilizer, 2022). On the other hand, the price of bush bean seeds kg^−1^ was obtained from the Humanitarian Data Exchange (2023). The labor costs (land preparation, planting, fertilizer application, weeding, pest, and disease management, and harvesting) were determined using the hourly rate of US$ 1.25 (Kenya Government, 2022). Bean yield was considered as the source of revenue. Return on investment, gross margin, and benefit cost ratio were calculated using Equations 4–7 (Sousa et al., 2022; Chia et al., 2019).


(4) Net income =Gross income−Total cost of production

(5) $$\text{Benefit cost ratio}=\frac{\text{Net income}}{\text{Total cost of production}}\;$$

(6) $$\text{Gross margin }\left(\%\right)=\frac{\text{Net income}}{\text{Gross income}}x\;100$$

(7) $$\text{Return on investment }\left(\%\right)=\frac{\text{Net income}}{\text{Total cost of production}}x\;100\;$$

### 2.5 Statistical analysis

Before analysis, data was checked for normality using the Shapiro-Wilk normality test. A linear mixed effect model from the package lme4, was used to analyze the number of leaves, leaf area, chlorophyll content, stem diameter, and plant height. During analysis, sampling time and fertilizer treatments were considered fixed effects, while replication was a random effect. The number of flowers, pods, pod length, number of seeds per pod, 100-seed weight, seed yield, haulm yield, N, P and K uptake, and quantity of N fixed were analyzed using one-way analysis of variance. The least-square means were computed using the ‘lsmeans’ package followed by mean separation using Tukey’s honestly significant difference (HSD), which was implemented using “cld” function from the “multicompView” package. To examine the relationship between bean growth and yield parameters, principal component analysis (PCA) was performed using “prcomp” function from “ggbiplot” packages. Data were analyzed separately for each season. The statistical analyses were conducted using R software version 4.3.0 (R Core Team, 2022).

## 3 Results

### 3.1 Influence of black soldier fly frass fertilizer and other commercial fertilizers on bush bean growth

#### 3.1.1 Number of leaves and leaf area

The number of leaves significantly varied at different growth stages due to the application of different fertilizer treatments (short rain season: χ2 = 2960.74, df = 14, p< 0.001, long rain season: χ2 = 3032.81, df = 14, p< 0.001) (**Table 2**). On the other hand, the number of leaves varied significantly at different growth stages during the study (short rain season: χ2 = 31385.07, df = 4, p< 0.001, long rain season: χ2 = 128019.82, df = 4, p< 0.001). The interaction effect between treatments and growth stages on the number of leaves was significant (χ2 = 713.85, df = 4, p< 0.001) during the long rain season only. The number of leaves increased to peak values (24 – 37 during short rains and 36 – 47 leaves during long rains) at the late vegetative stage (56 dap) and decreased afterward. Beans grown in soil amended with sole BSFFF at 45 kg N ha^−1^ had the highest number of leaves during both seasons, which were 3 – 5%, 3 – 18%, and 28 – 52% higher than the values achieved using sole NPK, equivalent rate of Phymyx, and control, respectively. Sole application of BSFFF at a rate of 45 kg N ha^−1^ significantly (p< 0.001) increased the number of leaves by 7 – 18% relative to the combined application of rhizobia inoculant and BSFFF at rates of 15 and 30 kg N ha^−1^.

**Table 2:** Number of leaves of bush beans as influenced by different fertilizer treatments.

Season	Treatment	Days after planting				
		14	28	42	56	70
Short rains (October 2022 – Jan 2023)	Control	5.2 ± 0.8b	10.7 ± 0.6g	22.4 ± 0.3f	24.1 ± 0.9h	17.5 ± 0.7g
	15BSFFF	5.6 ± 0.2ab	15.7 ± 0.7f	26.4 ± 0.9e	27.4 ± 0.7fg	19.5 ± 0.8efg
	30BSFFF	6.1 ± 0.3ab	17.9 ± 0.8cdef	28.7± 0.8bcd	29.6 ± 0.9bcdef	23.4 ± 0.9cd
	45BSFFF	6.9 ± 0.1a	20.4 ± 0.9ab	35.5 ± 1.1a	36.5 ± 1.7a	28.7 ± 1.1a
	15PHY	5.4 ± 0.8ab	12.6 ± 0.9g	23.5 ± 0.9f	25.3 ± 0.6gh	19.1 ± 0.9fg
	30PHY	6.0 ± 0.6ab	17.4 ± 0.9def	28.1 ± 0.8cde	29.3 ± 0.6cdef	22.3 ± 0.8d
	45PHY	6.6 ± 0.6ab	20.3 ± 1.0abc	30.4 ± 0.9b	31.1 ± 0.9bc	26.6 ± 0.9ab
	NPK	6.7 ± 0.6ab	22.3 ± 0.7a	34.0 ± 1.0a	35.5 ± 1.3a	28.4 ± 0.9a
	Rh+15BSFFF	5.7 ± 0.4ab	16.4 ± 0.6ef	27.0 ± 0.7de	28.7 ± 0.9def	21.6 ± 0.9de
	Rh+30BSFFF	6.6 ± 0.7ab	19.6 ± 0.9bcd	29.6 ± 0.6bc	30.5 ± 0.9bcd	26.5 ± 0.7ab
	Rh+45BSFFF	6.7 ± 0.3ab	20.3 ± 0.8ab	30.6 ± 1.0b	31.8 ± 1.0b	28.0 ± 0.8ab
	Rh+15PHY	5.7 ± 0.4ab	15.9 ± 0.4f	26.5 ± 0.8de	27.5 ± 0.7efg	21.4 ± 0.8def
	Rh+30PHY	6.6 ± 0.7ab	19.3 ± 1.0bcd	29.5 ± 0.9bc	30.0 ± 0.6bcd	26.4 ± 0.9ab
	Rh+45PHY	6.1 ± 0.2ab	18.6 ± 1.0bcde	29.4 ± 1.0bc	29.8 ± 0.6bcde	25.6 ± 1.0bc
	Rh+NPK	5.8 ± 0.6ab	16.6 ± 0.4ef	27.5 ± 1.0cde	28.9 ± 0.8cdef	21.7 ± 0.9de
	*Df* *F value* *p value*	*14* *2.87* *0.008*	*14* *47.04* *< 0.001*	*14* *62.52* *< 0.001*	*14* *50.84* *< 0.001*	*14* *57.44* *< 0.001*
Long rains (March 2023 – June 2023)	Control	5.9 ± 0.3a	17.9 ± 0.4j	35.4 ± 0.7g	36.3 ± 0.6g	35.1 ± 1.5d
	15BSFFF	5.9 ± 1.1a	20.6 ± 0.7hi	38.3 ± 1.0ef	38.6 ± 0.8f	36.0 ± 1.2d
	30BSFFF	6.3 ± 0.3a	23.1 ± 0.5def	39.9 ± 1.7bcde	42.2 ± 0.6d	39.3 ± 0.7abcd
	45BSFFF	7.1 ± 0.3a	26.7 ± 0.9ab	42.4 ± 1.9a	46.6 ± 1.9a	41.4 ± 0.6a
	15PHY	5.9 ± 0.6a	21.1 ± 0.6i	37.4 ± 0.8fg	36.4 ± 0.8g	35.8 ± 1.3d
	30PHY	6.3 ± 0.2a	22.8 ± 0.5efg	39.8 ± 0.9bcde	41.6 ± 1.0d	39.2 ± 0.4abcd
	45PHY	6.8 ± 0.2a	24.4 ± 0.7cd	40.4 ± 0.9bc	44.4 ± 0.7bc	40.8 ± 3.2ab
	NPK	7.1 ± 0.2a	28.1 ± 1.2a	41.4 ± 1.5ab	45.4 ± 1.7ab	41.1 ± 1.0a
	Rh+15BSFFF	6.1 ± 0.4a	21.6 ± 0.6ghi	38.5 ± 1.0def	39.4 ± 0.9ef	36.8 ± 0.4cd
	Rh+30BSFFF	6.4 ± 0.3a	24.2 ± 0.5cde	40.2 ± 0.8bc	43.6 ± 0.5bcd	40.2 ± 0.4abc
	Rh+45BSFFF	6.9 ± 0.5a	25.7 ± 0.8bc	41.3 ± 0.9ab	45.1 ± 1.4ab	41.0 ± 2.3ab
	Rh+15PHY	6.0 ± 0.2a	21.4 ± 0.6ghi	38.4 ± 0.8def	39.3 ± 0.7ef	36.5 ± 1.1cd
	Rh+30PHY	6.4 ± 0.3a	23.9 ± 0.4def	40.1 ± 1.1bcd	42.4 ± 1.0cd	39.5 ± 0.7abcd
	Rh+45PHY	6.3 ± 0.2a	23.3 ± 0.7def	40.0 ± 0.9bcd	42.3 ± 0.9cd	39.4 ± 0.6abcd
	Rh+NPK	6.1 ± 0.3a	22.5 ± 0.7fgh	39.3 ± 1.0cde	41.5 ± 0.8de	37.1 ± 0.9bcd
	*Df* *F/*χ2 *value* *p value*	*14* 42.13^*^ *0.086*	*14* *76.35* *< 0.001*	*14* *24.29* *< 0.001*	*14* *56.26* *< 0.001*	*14* *7.69* *< 0.001*

15BSFFF, 30 BSFFF, 45 BSFFF = application rates equivalent to 15, 30, and 45 kg N ha^−1^ of black soldier fly frass fertilizer; 15PHY, 30PHY, 45PHY = application rates equivalent to 15, 30, and 45 kg N ha^−1^ of Phymyx commercial organic fertilizer; NPK = Mineral fertilizer (Urea, DAP, MOP); Rh = rhizobia inoculant; Control = unfertilized plot, ^*^= χ2 value, Df = Degrees of freedom, F value = Fisher’s value, p = probability value. Per column, mean (± standard error) followed by the same letter(s) are not significantly different at p ≤ 0.05.

On the other hand, Phymyx applied alone at a rate of 45 kg N ha^−1^ resulted in a significant (p< 0.001) increase in the number of leaves as compared to when its rate of 15 kg N ha^−1^ and rhizobia inoculant were applied in combination. At 56 dap, the number of leaves significantly (p< 0.001) increased in plots treated with sole NPK (23%) and BSFFF rate of 45 kg N ha^−1^ (15%) compared to plots amended with rhizobia inoculant during the short rain season. However, during the long rain season, plots amended with rhizobia inoculant had an 8% higher (p< 0.001) number of leaves, compared to Phymyx applied at a rate of 15 kg N ha^−1^, while NPK caused a 9% significant increase compared with rhizobia inoculant.

The leaf area of bush beans varied significantly due to different fertilizer treatments (short rain season: χ2 = 6380.17, df = 14, p = 0.003, long rain season: χ2 = 11315.50, df = 14, p< 0.001), and at different growth stages (short rain season: χ2 = 65014.30, df = 4, p = p< 0.001, long rain season: χ2 = 255280.80, df = 4, p = p< 0.001) (**Table 3**). The interaction effect of fertilizer treatments and growth stages was not significant (short rain season: χ2 = 22.80, df = 56, p = 0.084, long rain season: χ2 = 10.72, df = 56, p =0.063). The leaf area increased throughout the experiments, to peak values of 37.8 – 52.7 cm^2^ and 90.7 – 124.9 cm^2^ during the short and long rain seasons, respectively at 70 dap (**Table 3**). The highest bean leaf area was achieved using sole BSFFF at a rate of 45 kg N ha^−1^, which was significantly (p< 0.001) higher than the control by 38%.

**Table 3:** Leaf area (cm^2^) of bush beans as influenced by different fertilizer treatments.

Season	Treatment	Days after planting				
		14	28	42	56	70
Short rains (October 2022 – January 2023)	Control	9.6 ± 0.8g	31.8 ± 1.6e	33.2 ± 2.0f	34.8 ± 1.8e	37.8 ± 1.0d
	15BSFFF	11.5 ± 0.8efg	35.6 ± 2.1de	39.3 ± 0.5e	39.6 ± 0.5de	41.1 ± 0.8cd
	30BSFFF	12.9 ± 0.3cde	40.7 ± 2.2bc	43.3 ± 0.8bcd	44.7 ± 1.9bc	45.6 ± 1.8bc
	45BSFFF	17.5 ± 1.1a	47.1 ± 2.1a	49.4 ± 2.3a	50.0 ± 1.9a	52.7 ± 4.6a
	15PHY	10.8 ± 0.5fg	37.5 ± 0.7cd	39.0 ± 0.4e	39.6 ± 0.4de	40.2 ± 0.5cd
	30PHY	13.0 ± 0.7cde	40.8 ± 0.3bc	41.9 ± 0.7cde	43.9 ± 0.4cd	45.6 ± 2.2bc
	45PHY	13.8 ± 0.6bc	44.2 ± 0.5ab	46.2 ± 1.1ab	49.1 ± 2.4ab	50.9 ± 2.7ab
	NPK	15.4 ± 0.7ab	46.6 ± 3.2a	48.8 ± 1.2a	49.6 ± 1.1a	51.0 ± 2.1ab
	Rh+15BSFFF	12.4 ± 0.9cdef	39.5 ± 0.6bcd	40.6 ± 0.5de	42.7 ± 1.2cd	45.1 ± 3.0bc
	Rh+30BSFFF	13.7 ± 0.5bcd	44.0 ± 0.6ab	45.9 ± 1.2ab	47.1 ± 1.7abc	49.6 ± 1.6ab
	Rh+45BSFFF	14.5 ± 0.9bc	46.4 ± 0.9a	48.6 ± 1.3a	49.5 ± 1.1ab	50.9 ± 1.8ab
	Rh+15PHY	11.6 ± 0.9defg	39.4 ± 0.7bcd	40.3 ± 1.2de	42.7 ± 2.8cd	45.0 ± 0.5bc
	Rh+30PHY	13.4 ± 0.9bcde	42.8 ± 3.2ab	44.9 ± 0.6bc	46.6 ± 1.2abc	48.8 ± 1.2ab
	Rh+45PHY	13.1 ± 0.5cde	44.2 ± 0.9ab	46.1 ± 1.1ab	46.7 ± 1.7abc	48.0 ± 1.9ab
	Rh+NPK	12.5 ± 0.9cdef	41.4 ± 0.7bc	44.1 ± 1.0bc	46.8 ± 1.8abc	47.7 ± 1.3ab
	*Df* *F value* *p value*	*14* *21.97* *< 0.001*	*14* *20.64* *< 0.001*	*14* *43.09* *< 0.001*	*14* *22.48* *< 0.001*	*14* *13.24* *< 0.001*
Long rains (March 2023 – June 2023)	Control	13.4 ± 1.9c	67.3 ± 11.0b	82.5 ± 4.2b	89.0 ± 4.6a	90.7 ± 4.2b
	15BSFFF	14.3 ± 0.9c	68.0 ± 11.6b	86.4 ± 3.4ab	98.5 ± 10.0a	102.5 ± 3.1ab
	30BSFFF	15.6 ± 0.5c	77.4 ± 1.7ab	89.4 ± 8.2ab	107.8 ± 8.4a	111.5 ± 5.6ab
	45BSFFF	21.2 ± 3.1a	116.3 ± 7.6a	119.1 ± 5.8a	123.8 ± 4.4a	124.9 ± 4.4a
	15PHY	13.9 ± 1.0c	67.8 ± 4.1b	85.4 ± 4.8ab	101.4 ± 3.4a	102.3 ± 9.8ab
	30PHY	15.5 ± 0.7bc	76.5 ± 3.9ab	90.5 ± 9.8ab	109.4 ± 1.6a	109.9 ± 11.3ab
	45PHY	16.7 ± 0.6abc	84.1 ± 5.1ab	104.8 ± 13.1ab	111.4 ± 7.7a	112.7 ± 7.7ab
	NPK	19.4 ± 0.6ab	106.4 ± 6.8ab	118.2 ± 3.0a	121.9 ± 6.9a	124.3 ± 6.5ab
	Rh+15BSFFF	15.3 ± 0.6bc	74.7 ± 5.3ab	87.6 ± 7.0ab	103.4 ± 3.0a	109.4 ± 1.3ab
	Rh+30BSFFF	16.6 ± 0.5abc	83.7 ± 5.6ab	104.2 ± 6.7ab	111.1 ± 5.6a	112.7 ± 5.2ab
	Rh+45BSFFF	16.9 ± 0.5abc	93.6 ± 18.6ab	109.3 ± 7.6ab	111.9 ± 7.8a	113.8 ± 3.5ab
	Rh+15PHY	14.9 ± 0.9bc	71.6 ± 11.3ab	87.5 ± 5.0ab	106.7 ± 9.7a	107.8 ± 2.6ab
	Rh+30PHY	16.1 ± 0.9bc	80.5 ± 9.3ab	103.4 ± 3.0ab	111.1 ± 5.2a	112.2 ± 7.4ab
	Rh+45PHY	15.9 ± 0.3bc	79.8 ± 4.8ab	98.5 ± 5.6ab	109.9 ± 6.2a	110.3 ± 6.5ab
	Rh+NPK	15.4 ± 0.9bc	76.9 ± 6.6ab	89.2 ± 1.6ab	108.6 ± 11.8a	109.4 ± 9.3ab
	*Df* *F value* *p value*	*14* *4.77* *< 0.001*	*14* *2.62* *0.013*	*14* *3.35* *0.003*	*14* *1.47* *0.181*	*14* *1.74* *0.100*

15BSFFF, 30 BSFFF, 45 BSFFF = application rates equivalent to 15, 30, and 45 kg N ha^−1^ of black soldier fly frass fertilizer; 15PHY, 30PHY, 45PHY = application rates equivalent to 15, 30, and 45 kg N ha^−1^ of Phymyx commercial organic fertilizer; NPK = Mineral fertilizer (Urea, DAP, MOP); Rh = rhizobia inoculant; Control = unfertilized plot. Per column, mean (± standard error) followed by the same letter(s) are not significantly different at p ≤ 0.05.

#### 3.1.2 Leaf chlorophyll content

The leaf chlorophyll content varied significantly due to different fertilizer treatments (short rain season: χ2 = 1571.57, df = 14, p< 0.001, long rain season: χ2 = 1158.69, df = 14, p< 0.001) (**Table 4**). Additionally, the growth stages significantly influenced the chlorophyll content of bush beans during the short rain season (χ2 = 908.35, df = 4, p< 0.001) and long rain season (χ2 = 1158.69, df = 4, p< 0.001). The interaction effect between fertilizer treatments and growth stages was significant (short rain season: χ2 = 437.36, df = 56, p< 0.001, long rain season: χ2 = 533.45, df = 56, p< 0.001). Chlorophyll content reached peak values at the late vegetative stage (56 dap) during the short and long rain seasons. Thereafter, the chlorophyll content followed a decreasing trend (**Table 4**).

**Table 4:** Chlorophyll content (SPAD values) of bush beans as influenced by different fertilizer treatments.

Season	Treatment	Days after planting				
		14	28	42	56	70
Short rains (October 2022 – January 2023)	Control	29.0 ± 0.6c	30.8± 1.4d	31.7 ± 0.8f	33.0 ± 1.5d	31.5 ± 2.0c
	15BSFFF	30.7 ± 0.9abc	31.0 ± 0.8d	31.5 ± 1.0f	33.6 ± 1.0cd	32.4 ± 0.8bc
	30BSFFF	31.7 ± 1.5abc	32.5 ± 1.0bcd	34.7 ± 0.9cde	36.4 ± 0.9abcd	33.6 ± 0.9abc
	45BSFFF	32.9 ± 1.9a	35.2 ± 1.2a	39.1 ± 1.0a	40.0 ± 1.6a	35.7 ± 2.1a
	15PHY	29.1 ± 0.9bc	30.4 ± 1.3d	30.7 ± 1.0f	33.5 ± 1.1cd	31.5 ± 1.0c
	30PHY	31.6 ± 0.8abc	32.6 ± 0.9abcd	34.6 ± 0.9cde	36.3 ± 1.0abcd	33.4 ± 1.2abc
	45PHY	32.3 ± 1.0a	34.2 ± 1.2abc	36.6 ± 0.8bc	37.7 ± 0.8ab	34.6 ± 0.9ab
	NPK	32.7 ± 0.9a	35.1 ± 0.9ab	38.6 ± 1.1ab	39.4 ± 0.9a	35.7 ± 0.9a
	Rh+15BSFFF	31.3 ± 1.0abc	31.6 ± 0.7cd	34.3 ± 0.9de	34.9 ± 0.9bcd	33.2 ± 0.8abc
	Rh+30BSFFF	32.2 ± 1.0ab	32.9 ± 1.0abcd	35.6 ± 0.9cd	37.5 ± 1.0ab	34.5 ± 1.1ab
	Rh+45BSFFF	32.6 ± 2.5a	33.4 ± 0.9abcd	38.5 ± 1.0ab	39.3 ± 1.0a	35.3 ± 0.8ab
	Rh+15PHY	31.0 ± 0.7abc	31.7 ± 0.6cd	32.5 ± 0.9ef	34.4 ± 0.8bcd	33.1 ± 0.9abc
	Rh+30PHY	31.9 ± 0.9abc	32.8± 1.0abcd	35.4 ± 1.0cd	37.4 ± 0.8ab	34.4 ± 0.7ab
	Rh+45PHY	31.8 ± 0.9abc	32.7 ± 0.9abcd	35.0 ± 1.0cd	36.7 ± 0.8abc	33.7 ± 0.5abc
	Rh+NPK	31.5 ± 1.0abc	32.4 ± 1.1bcd	34.4 ± 0.9cde	35.4 ± 0.9bcd	33.3 ± 1.0abc
	*Df* *F value* *p value*	*14* *3.86* *0.001*	*14* *37.13* *< 0.001*	*14* *6.53* *< 0.001*	*14* *10.28* *< 0.001*	*14* *5.65* *< 0.001*
Long rains (March 2023 – June 2023)	Control	30.7 ± 0.6c	32.1 ± 0.8e	33.0 ± 0.3f	36.3 ± 0.6g	35.4 ± 1.0i
	15BSFFF	31.3 ± 0.5bc	32.6 ± 0.6cde	33.8 ± 0.9def	38.6 ± 0.6f	37.5 ± 0.9ghi
	30BSFFF	31.9 ± 0.6abc	33.3 ± 0.5abcde	35.4 ± 0.6bcd	42.2 ± 0.5d	39.9 ± 0.5de
	45BSFFF	33.7 ± 1.5a	35.6 ± 0.5a	37.9 ± 0.7a	46.6 ± 1.1a	45.5 ± 0.7a
	15PHY	31.2 ± 0.9bc	32.2 ± 0.8de	33.4 ± 0.7ef	36.4 ± 0.7g	36.1 ± 0.7hi
	30PHY	31.7 ± 0.5abc	33.1 ± 0.6abcde	35.3 ± 0.9bcd	41.6 ± 0.9d	39.8 ± 0.9def
	45PHY	32.7 ± 1.0ab	33.7 ± 0.6abc	36.0 ± 0.9bc	44.4 ± 0.7bc	42.5 ± 0.9bc
	NPK	33.0 ± 1.3ab	35.2 ± 0.8ab	36.7 ± 0.8ab	45.4 ± 0.8ab	43.2 ± 0.8b
	Rh+15BSFFF	31.5 ± 0.7abc	32.8 ± 0.4bcde	35.5 ± 0.6cdef	39.4 ± 0.9ef	38.3 ± 0.6efg
	Rh+30BSFFF	32.4 ± 0.8abc	33.6 ± 0.7abcd	35.9 ± 0.7bc	43.6 ± 0.5bcd	42.4 ± 0.7bc
	Rh+45BSFFF	32.8 ± 1.3ab	33.9 ± 1.0abc	36.6 ± 0.5ab	45.1 ± 0.9ab	43.1 ± 1.0bc
	Rh+15PHY	31.4 ± 0.6bc	32.7 ± 0.4cde	34.4 ± 0.9cdef	39.3 ± 0.7ef	37.7 ± 0.9fgh
	Rh+30PHY	32.3 ± 0.7abc	33.5 ± 0.7abcde	35.7 ± 0.7bc	42.4 ± 0.8cd	41.1 ± 0.7bcd
	Rh+45PHY	32.0 ± 0.6abc	33.4 ± 0.8abcde	35.6 ± 0.5bcd	42.3 ± 1.0cd	41.0 ± 0.7cd
	Rh+NPK	31.6 ± 0.6abc	33.0 ± 0.4abcde	35.1 ± 0.6bcde	41.5 ± 0.8de	39.3 ± 0.9defg
	*Df* *F value* *p value*	*14* *4.10* *0.001*	*14* *6.06* *< 0.001*	*14* *13.69* *< 0.001*	*14* *56.26* *< 0.001*	*14* *48.60* *< 0.001*

15BSFFF, 30 BSFFF, 45 BSFFF = application rates equivalent to 15, 30, and 45 kg N ha^−1^ of black soldier fly frass fertilizer; 15PHY, 30PHY, 45PHY = application rates equivalent to 15, 30, and 45 kg N ha^−1^ of Phymyx commercial organic fertilizer; NPK = Mineral fertilizer (Urea, DAP, MOP); Rh = rhizobia inoculant; Control = unfertilized plot. Per column, means (± standard error) followed by the same letter(s) are not significantly different at p ≤ 0.05. Df = Degrees of freedom, F value = Fisher’s value, p = probability value. Df = Degrees of freedom, F value = Fisher’s value, p = probability value.

Plots treated with sole BSFFF rate of 45 kg N ha^−1^ produced beans with the highest chlorophyll content, which was significantly (p< 0.001) higher than the values achieved under control and using an equivalent rate of Phymyx by 28 and 5%, respectively. Combined application of rhizobia inoculant and Phymyx at a rate of 15 kg N ha^−1^ significantly increased chlorophyll by 8% compared to the sole application of Phymyx Also, a combined application of BSFFF rates of 15 and 30 kg N ha^−1^ and rhizobia increased (p< 0.001) chlorophyll content compared to sole application of BSFFF at the rate of 45 kg N ha^−1^. Chlorophyll content at 56 dap was significantly (p< 0.001) higher (11%) in plots treated with NPK than those under inoculation during the short rain season. On the other hand, plots amended with rhizobia inoculant recorded (p< 0.001) higher chlorophyll content (8%) than the plots of Phymyx at 15 kg N ha^−1^, whereas NPK plots performed better (9%) than rhizobia inoculant, during the long rain season.

#### 3.1.3 Plant height

Different fertilizer treatments significantly influenced the heights of bush beans during the short rain season (χ2 = 8152.80, df = 14, p< 0.001) and long rain season (χ2 = 2254.00, df = 14, p< 0.001) (**Table 5**). The plant height also varied significantly at different growth stages (short rain season: χ2 = 58904.80, df = 4, p< 0.001, long rain season: χ2 = 138910.40, df = 4, p< 0.001). The interaction effect of growth stages and fertilizer treatments was significant during the long rain season only (χ2 = 429.40, df = 56, p< 0.001). There was a sharp rise in plant height between 14 dap and 28 dap during both seasons. Sole application of BSFFF at a rate of 45 kg N ha^−1^ produced beans with the tallest plants, which were 15% and 6% higher compared to the control and equivalent rate of Phymyx, respectively. For Phymyx, significant (p< 0.001) increases in plant height above that attained in the control were achieved at the rate of 45 kg N ha^−1^. At 70 dap, bean plants were significantly (p< 0.001) taller in plots under inoculation (13%) compared with those treated with BSFFF at 15 kg N ha^−1^. Also, an increase in plant height was noted in plots amended with BSFFF at 45 kg N ha^−1^ (11%) and NPK (19%) compared with those treated with rhizobia inoculant, during the short rain season. During the long rain season (70 dap), a significant (p< 0.001) increase in plant height was only recorded in plots treated with NPK (7%) compared with those amended with rhizobia inoculant.

**Table 5:** Plant height of bush beans as influenced by different fertilizer treatments.

Season	Treatment	Days after planting				
		14	28	42	56	70
Short rains (October 2022 – January 2023)	Control	7.7 ± 1.30d	26.0 ± 1.41h	28.5 ± 2.1f	29.4 ± 2.7e	29.8 ± 1.2g
	15BSFFF	7.8 ± 0.9cd	29.3 ± 0.5efg	30.3 ± 0.4f	30.4 ± 0.8e	31.8 ± 1.0g
	30BSFFF	9.8 ± 0.8abcd	32.0 ± 0.7de	36.3 ± 0.8cde	37.5 ± 0.6bcd	38.6 ± 1.2def
	45BSFFF	11.5 ± 1.0a	39.3 ± 0.4a	42.1 ± 1.1a	44.2 ± 1.5a	46. 3 ± 1.4a
	15PHY	7.7 ± 1.1cd	27.6 ± 0.8gh	29.5 ± 0.8f	30.4 ± 0.9e	30.9 ± 0.9g
	30PHY	9.2 ± 0.6abcd	31.6 ± 0.9de	35.5 ± 0.6de	36.4 ± 0.7cd	36.9 ± 0.9ef
	45PHY	10.3 ± 0.5ab	35.6 ± 0.6bc	38.5 ± 0.5bc	39.8 ± 0.8bc	40.1 ± 0.7cd
	NPK	11.5 ± 0.9a	37.3 ± 2.3ab	41.9 ± 0.4a	43.7 ± 1.4a	43.6 ± 1.2ab
	Rh+15BSFFF	8.4 ± 1.0bcd	30.4 ± 0.9efg	34.6 ± 0.8e	35.7 ± 1.1d	36.0 ± 0.8f
	Rh+30BSFFF	10.1 ± 0.6abc	35.4 ± 0.8bc	37.8 ± 0.6bcd	39.0 ± 0.5bcd	40.1 ± 0.9cd
	Rh+45BSFFF	10.6 ± 0.6ab	35.9 ± 0.9bc	39.9 ± 1.36ab	40.8 ± 0.7ab	41.9 ± 0.8bc
	Rh+15PHY	8.4 ± 0.7bcd	28.6 ± 0.9fgh	30.8 ± 0.8f	31.4 ± 1.0e	31.8 ± 0.8g
	Rh+30PHY	10.0 ± 0.5abcd	35.3 ± 0.5bc	37.5 ± 0.6bcde	38.0 ± 0.7bcd	38.4 ± 0.9def
	Rh+45PHY	9.9 ± 0.4abcd	33.6 ± 0.5cd	36.4 ± 1.1cde	38.1 ± 0.7bcd	39.3 ± 0.9cde
	Rh+NPK	9.2 ± 0.8abcd	30.59 ± 0.9ef	35.2 ± 1.4de	35.8 ± 1.5d	36.5 ± 1.3ef
	*Df* *F value* *p value*	*14* *5.47* *< 0.001*	*14* *49.26* *< 0.001*	*14* *56.51* *< 0.001*	*14* *49.78* *< 0.001*	*14* *70.23* *< 0.001*
Long rains (March 2023 – June 2023)	Control	9.6 ± 0.6c	32.0 ± 1.7f	37.4 ± 1.0f	48.1 ± 1.3d	48.5 ± 1.4f
	15BSFFF	10.4 ± 0.6bc	32.4 ± 0.9ef	39.4 ± 0.7ef	48.6 ± 0.9d	48.8 ± 1.0ef
	30BSFFF	10.9 ± 0.6abc	33.9 ± 0.8cdef	41.5 ± 0.6cde	50.0 ± 0.6cd	50.5 ± 0.8def
	45BSFFF	13.0 ± 1.7a	37.3 ± 1.5a	46.9 ± 1.0a	54.0 ± 1.5a	55.6 ± 1.5a
	15PHY	11.0 ± 1.6abc	33.2 ± 1.1def	39.3 ± 0.9ef	48.5 ± 1.1d	48.7 ± 1.2ef
	30PHY	10.8 ± 0.5abc	33.8 ± 0.7cdef	41.0 ± 0.9cde	49.8 ± 0.6cd	50.4 ± 0.6def
	45PHY	11.5 ± 0.6abc	35.6 ± 0.5abcd	43.2 ± 0.7bc	52.0 ± 0.7abc	52.4 ± 0.7bcd
	NPK	12.6 ± 1.1ab	36.5 ± 0.6ab	46.5 ± 0.8a	53.3 ± 0.9a	53.9 ± 0.6ab
	Rh+15BSFFF	10.5 ± 0.6abc	33.4 ± 1.0cdef	40.4 ± 0.6de	48.9 ± 0.9d	49.3 ± 0.9ef
	Rh+30BSFFF	11.1 ± 0.8abc	35.5 ± 0.8abcd	42.6 ± 0.5cd	51.9 ± 0.7abc	52.3 ± 0.8bcd
	Rh+45BSFFF	12.2 ± 0.7ab	35.7 ± 0.8abc	45.4 ± 0.8ab	52.9 ± 0.6ab	53.3 ± 1.2abc
	Rh+15PHY	10.6 ± 0.6abc	33.4 ± 0.7def	39.7 ± 1.0ef	48.6 ± 0.7d	49.4 ± 0.6ef
	Rh+30PHY	11.04 ± 0.5abcd	34.7 ± 1.0bcd	41.7 ± 1.1cde	50.5 ± 0.8bcd	51.1 ± 1.0cde
	Rh+45PHY	11.00 ± 0.8abcd	34.4 ± 1.0bcde	41.5 ± 0.9cde	50.3 ± 1.0bcd	50.6 ± 1.0def
	Rh+NPK	10.7 ± 1.2abc	33.5 ± 0.6cdef	41.2 ± 1.2cde	49.6 ± 0.8cd	50.3 ± 0.9def
	*Df* *F/χ2 value* *p value*	*14* 45.23^*^ *0.003*	*14* *11.21* *< 0.001*	*14* *32.05* *< 0.001*	*14* *13.30* *< 0.001*	*14* *19.43* *< 0.001*

15BSFFF, 30 BSFFF, 45 BSFFF = application rates equivalent to 15, 30, and 45 kg N ha^−1^ of black soldier fly frass fertilizer; 15PHY, 30PHY, 45PHY = application rates equivalent to 15, 30, and 45 kg N ha^−1^ of Phymyx commercial organic fertilizer; NPK = Mineral fertilizer (Urea, DAP, MOP); Rh = rhizobia inoculant; Control = unfertilized plot, ^*^= χ2 value. Df = Degrees of freedom, F value = Fisher’s value, p = probability value Df = Degrees of freedom, F value = Fisher’s value, p = probability value. Per column, means (± standard error) followed by the same letter(s) are not significantly different at p ≤ 0.05.

#### 3.1.4 Stem diameter

Across the seasons, the bean stem diameter varied significantly due to different fertilizer applications (short rain season: χ2 = 420.07, df = 14, p< 0.001, long rain season: χ2 = 269.11, df = 14, p< 0.001) (**Table 6**). Also, the stem diameter varied significantly (p< 0.001) across the growth stages during the short rain season (χ2 = 343.75, df = 4, p< 0.001) and long rain season (χ2 = 1553.42, df = 4, p< 0.001). The interaction effect between the fertilizer treatments and growth stages was not significant during both seasons (short rain season: χ2 = 13.65, df = 56, p = 0.918, long rain season: χ2 = 25.92, df = 56, p = 0.999).

**Table 6:** Bean stem diameter of bush beans as influenced by different fertilizer treatments.

Season	Treatment	Days after planting				
		14	28	42	56	70
Short rains (October 2022 – January 2023)	Control	3.2 ± 0.09d	3.6 ± 0.3e	3.7 ± 0.3d	4.3 ± 0.04d	4.4 ± 0.2c
	15BSFFF	3.6 ± 0.3cd	4.0 ± 0.1cde	4.1 ± 0.05cd	4.5 ± 0.2cd	4.7 ± 0.5bc
	30BSFFF	4.0 ± 0.2bc	4.3 ± 0.2abcd	4.6 ± 0.2abc	4.8 ± 0.4abc	4.9 ± 0.5abc
	45BSFFF	4.5 ± 0.07ab	4.8 ± 0.02ab	5.0 ± 0.1a	5.2 ± 0.1a	5.4 ± 0.1ab
	15PHY	3.5 ± 0.2cd	3.9 ± 0.1de	4.1 ± 0.06cd	4.9 ± 0.1abc	4.9 ± 0.3abc
	30PHY	3.9 ± 0.08bcd	4.2 ± 0.05bcde	4.6 ± 0.3abc	4.8 ± 0.2abc	5.3 ± 0.4ab
	45PHY	4.1 ± 0.1abc	4.6 ± 0.4abc	4.7 ± 0.2abc	5.1 ± 0.3abc	5.2 ± 0.3ab
	NPK	4.8 ± 0.2a	4.9 ± 0.2a	5.1 ± 0.09a	5.4 ± 0.2a	5.5 ± 0.1a
	Rh+15BSFFF	3.9 ± 0.2bcd	4.1 ± 0.08cde	4.3 ± 0.02bcd	4.6 ± 0.3bcd	4.9 ± 0.2abc
	Rh+30BSFFF	4.1 ± 0.1abc	4.5 ± 0.3abcd	4.7 ± 0.1abc	5.0 ± 0.02abc	5.1 ± 0.03ab
	Rh+45BSFFF	4.4 ± 0.2ab	4.6 ± 0.2abc	4.9 ± 0.1ab	5.1 ± 0.05abc	5.2 ± 0.06ab
	Rh+15PHY	3.8 ± 0.7bcd	4.0 ± 0.1cde	4.2 ± 0.3cd	4.5 ± 0.09cd	4.8 ± 0.1abc
	Rh+30PHY	4.0 ± 0.2bc	4.4 ± 0.3abcd	4.7 ± 0.2abc	5.0 ± 0.1abc	5.0 ± 0.1ab
	Rh+45PHY	4.0 ± 0.09bc	4.4 ± 0.2abcd	4.6 ± 0.4abc	4.8 ± 0.3abc	4.9 ± 0.1abc
	Rh+NPK	3.9 ± 0.09bcd	4.2 ± 0.2bcde	4.3 ± 0.2bcd	4.7 ± 0.2bcd	4.8 ± 0.2bc
	*Df* *F value* *p value*	*14* *7.42* *< 0.001*	*14* *8.11* *< 0.001*	*14* *9.68* *< 0.001*	*14* *6.34* *< 0.001*	*14* *4.54* *< 0.001*
Long rains (March 2023 – June 2023)	Control	3.5 ± 0.2c	3.9 ± 0.2e	4.9 ± 0.3c	5.6 ± 0.4c	5.7 ± 0.5c
	15BSFFF	3.7 ± 0.1bc	4.3 ± 0.3cde	5.3 ± 0.2bc	6.1 ± 0.07bc	6.1 ± 0.6bc
	30BSFFF	4.0 ± 0.5abc	4.9 ± 0.1abcd	5.8 ± 0.5abc	6.7 ± 0.4abc	6.8 ± 0.6abc
	45BSFFF	4.2 ± 0.1a	5.5 ± 0.4ab	6.4 ± 0.6ab	7.4 ± 0.6ab	7.5 ± 0.6ab
	15PHY	3.7 ± 0.3bc	4.2 ± 0.4de	5.2 ± 0.1bc	6.0 ± 0.04bc	6.1 ± 0.3bc
	30PHY	3.9 ± 0.08abc	4.8 ± 0.3bcde	5.6 ± 0.4abc	6.5 ± 0.6abc	6.6 ± 0.7abc
	45PHY	4.1 ± 0.2ab	5.1 ± 0.2abc	6.2 ± 0.3abc	6.9 ± 0.3abc	6.9 ± 0.4abc
	NPK	4.2 ± 0.1a	5.7 ± 0.5a	6.9 ± 0.5a	7.5 ± 0.4a	7.6 ± 0.4a
	Rh+15BSFFF	3.8 ± 0.1abc	4.7 ± 0.3bcde	5.5 ± 0.5bc	6.4 ± 0.7abc	6.4 ± 0.9abc
	Rh+30BSFFF	4.1 ± 0.1ab	5.0 ± 0.4abcd	6.1 ± 0.1abc	6.8 ± 0.2abc	6.8 ± 0.2abc
	Rh+45BSFFF	4.1 ± 0.4ab	5.2 ± 0.3abc	6.2 ± 0.2ab	7.3 ± 0.4ab	7.4 ± 0.3ab
	Rh+15PHY	3.8 ± 0.2abc	4.7 ± 0.4bcde	5.3 ± 0.3bc	6.4 ± 0.7abc	6.5 ± 0.6abc
	Rh+30PHY	4.0 ± 0.1ab	4.9 ± 0.4abcd	5.9 ± 0.2abc	6.6 ± 0.5abc	6.8 ± 0.5abc
	Rh+45PHY	3.9 ± 0.1abc	4.9 ± 0.2abcd	5.9 ± 0.4abc	6.7 ± 0.4abc	6.8 ± 0.6abc
	Rh+NPK	3.8 ± 0.2abc	4.8 ± 0.4bcde	5.5 ± 0.3bc	6.4 ± 0.6abc	6.6 ± 0.4abc
	*Df* *F value* *P value*	*14* *5.34* *< 0.001*	*14* *7.39* *< 0.001*	*14* *4.37* *< 0.001*	*14* *3.59* *0.002*	*14* *3.66* *0.001*

15BSFFF, 30 BSFFF, 45 BSFFF = application rates equivalent to 15, 30, and 45 kg N ha^−1^ of black soldier fly frass fertilizer; 15PHY, 30PHY, 45PHY = application rates equivalent to 15, 30, and 45 kg N ha^−1^ of Phymyx commercial organic fertilizer; NPK = Mineral fertilizer (Urea, DAP, MOP); Rh = rhizobia inoculant; Control = unfertilized plot. Per column, means (± standard error) followed by the same letter(s) are not significantly different at p ≤ 0.05. Df = Degrees of freedom, F value = Fisher’s value, p = probability value.

Stem diameter gradually increased to the highest values at 70 dap (**Table 6**). Beans that were grown using sole BSFFF at a rate of 45 kg N ha^−1^ and NPK had 32 and 33% significantly (p< 0.001) higher stem diameters, respectively, compared to the control treatment. Treating soils with NPK resulted in a 15% (p< 0.001) increase in bean stem diameter compared with plots amended with rhizobia inoculant during the short rain season.

### 3.2 Influence of various fertilizer treatments on nutrient uptake

**Figure 2:**
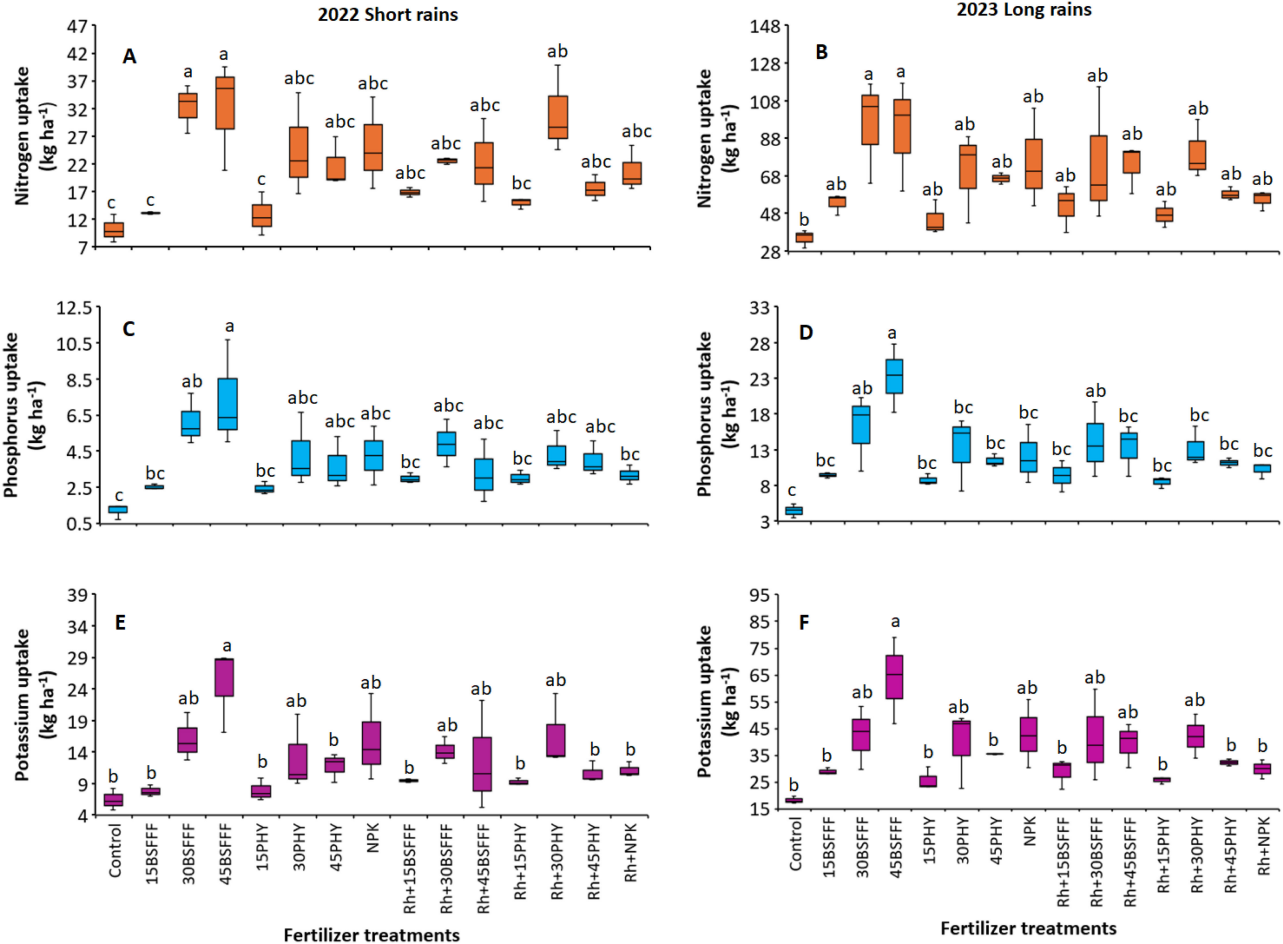
Nitrogen uptake **(A, B)**, Phosphorus uptake **(C, D)** and potassium uptake **(E, F)** of bush beans as influenced by different fertilizer treatments during the short rains and long rains seasons.15BSFFF, 30 BSFFF, 45 BFFF = application rates equivalent to 15, 30, and 45 kg N ha^−1^ of black soldier fly frass fertilizer; 15PHY, 30PHY, 45PHY = application rates equivalent to 15, 30, and 45 kg N ha^−1^ of Phymyx commercial organic fertilizer; NPK = Mineral fertilizer (Urea, DAP, MOP); Rh = rhizobia inoculant; Control = unfertilized plot. Per panel, means (± standard error) followed by the same letter(s) are not significantly different at *p* ≤ 0.05.

The different fertilizer treatments significantly influenced the nitrogen (N) uptake of bush beans during the short rain season (χ2 = 68.72, df = 14, p< 0.001) (**Figure 2A**) and long rain season (χ2 = 36.65, df = 14, p =< 0.001) (**Figure 2B**). The highest N uptake was observed in bush beans grown using sole BSFFF at a rate of 30 kg N ha^−1^ during the short rain season (32.4 kg N ha^−1^) and long rain season (95.4 kg N ha^−1^), which were 217% (short rain season) and 172% (long rain season) significantly (p< 0.001) higher than the N accumulated by bush beans in the control plots (**Figure 2B**). Additionally, bush beans grown in plots that had sole BSFFF applied at a rate of 30 kg N ha^−1^ accumulated a significantly (p< 0.001) higher N compared to sole application of either Phymyx or BSFFF at a rate of 15 kg N ha^−1^ during the short rain season. During the short rain season, sole BSFFF applied at a rate of 30 kg N ha^−1^ caused significantly (p< 0.001) higher N uptake compared to combined application of rhizobia inoculant and Phymyx at a rate of 15 kg N ha^−1^.

The P uptake of bush beans was also significantly influenced by the different fertilizer treatments (short rain season: χ2 = 52.89, df = 14, p< 0.001 (**Figure 2C**), long rain season: χ2 = 72.11, df = 14, p< 0.001) (**Figure 2D**). The highest P uptake was achieved in beans grown using sole BSFFF supplied at 45 kg N ha^-1^ during the short rain season and long rain season, and these were significantly (p< 0.001) higher than the value achieved using the control by 3 – 9 folds. Without inoculation, the application rate of 45 kg N ha^-1^ supplied as BSFFF caused 74% significantly (p< 0.001) higher P uptake compared to when rhizobia inoculant was combined with the treatment during the long rain season. Furthermore, the application of BSFFF singly at the rate of 45 kg N ha^-1^ boosted P uptake by 91% and 102% (p< 0.001) compared to sole NPK and the equivalent rate of sole Phymyx, respectively, during the long rain season. On the other hand, the application of sole BSFFF at a rate of 45 kg N ha^-1^ caused significantly (p< 0.001) higher P uptake compared to NPK applied in combination with rhizobia inoculant (131%), and Phymyx and BSFFF applied at a rate of 15 kg N ha^-1^ with or without rhizobia inoculant during the short rain season. There was a 74% (p< 0.001) increase in P uptake in plots amended with a BSFFF rate of 45 kg N ha^-1^ compared with plots treated with rhizobia inoculant during the long rain season.

There were significant variations in K uptake of bush beans due to different fertilizer treatments during the study (short rain season: χ2 = 50.17, df = 14, p< 0.001, long rain season: χ2 = 57.28, df = 14, p< 0.001) (**Figures 2E, F**). The highest K uptake was achieved using sole BSFFF at 45 kg N ha^-1^, and this was 250 – 287%, 79 – 112%, 114 – 123%, and 79 – 133% higher compared to the values obtained using the control, equivalent rate of Phymyx, combined application of NPK and rhizobia inoculant, and equivalent rate of Phymyx combined with rhizobia inoculant, respectively. Additionally, the sole application of BSFFF at a rate of 45 kg N ha^-1^ significantly enhanced K uptake relative to Phymyx and BSFFF applied at 15 kg N ha^-1^.

### 3.3 Biological nitrogen fixation of bush beans grown using BSF frass fertilizer and other commercial fertilizers

#### 3.3.1 Total and effective root nodules

The different fertilizer treatments caused significant variations in the total number of root nodules during the short rain season (F = 16.87, df = 14, p< 0.001) and long rain season (F = 31.61, df = 14, p< 0.001) (**Table 7**). Plots treated with sole BSFFF at a rate of 30 kg N ha^−1^ produced beans with the highest number of root nodules (30 – 36), which were 218% – 276%, 80 – 108%, 44 – 97%, and 36 – 71% higher compared to the control treatment, equivalent rate of Phymyx, sole NPK and combined application of NPK and rhizobia, respectively. The total number of root nodules achieved using sole BSFFF at a rate of 30 kg N ha^−1^ was 102 – 130% higher (p<0.001) compared to the integrated use of the fertilizer with rhizobia inoculant. On the contrary, combined application of rhizobia inoculant and Phymyx at a rate of 30 kg N ha^−1^ caused a significant (p< 0.001) increase in the number of root nodules (73 – 77%) compared to sole application of the same Phymyx treatment. The total number of root nodules (p< 0.001) increased due to the application of rhizobia inoculant compared to when Phymyx at 30 kg N ha^−1^ was applied during the short (77%) and long (73%) rain seasons. On the other hand, the BSFFF rate of 45 kg N ha^−1^ application led to a 56% significant (p< 0.001) increase in the total number of nodules compared with plots treated with rhizobia inoculant, during the long rain season.

**Table 7:** Total and effective root nodules, and amount of nitrogen fixed by bush beans grown in soil amended with different fertilizer treatments.

Treatment	2022 Short rains			2023 Long rains		
	Total root nodules plant^−1^	Effective root nodules plant^−1^	Nitrogen fixed (kg ha^−1^)	Total root nodules plant^−1^	Effective root nodules plant^−1^	Nitrogen fixed (kg ha^−1^)
Control	9.5 ± 1.2e	3.8 ± 0.7f	-2.2 ± 1.4c	9.6 ± 2.1f	5.2 ± 1.1f	22.7 ± 2.8c
15BSFFF	10.8 ± 1.8de	6.6 ± 2.2def	0.8 ± 0.1c	11.8 ± 1.2ef	8.0 ± 1.0def	41.2 ± 3.2ab
30BSFFF	30.3 ± 0.5a	17.2 ± 1.3a	20.1 ± 2.6a	36.1 ± 2.3a	19.2 ± 1.3a	82.9 ± 8.5a
45BSFFF	21.1 ± 1.7bc	15.8 ± 2.2abc	19.8 ± 5.7a	27.9 ± 0.9bc	17.2 ± 1.8ab	80.3 ± 6.9ab
15PHY	9.9 ± 2.4e	6.9 ± 1.4ef	0.4 ± 2.3c	10.0 ± 1.6ef	6.0 ± 0.8ef	32.5 ± 5.4bc
30PHY	14.6 ± 0.2cde	11.2 ± 0.3abcdef	12.4 ± 5.4abc	20.1 ± 1.7cd	13.9 ± 1.1abcd	58.2 ± 4.9abc
45PHY	18.4 ± 0.3bcd	12.4 ± 1.1abcde	9.5 ± 2.6abc	25.2 ± 2.1cd	14.7 ± 1.1abcd	54.2 ± 1.7abc
NPK	21.0 ± 1.3bc	15.7 ± 2.0abc	12.9 ± 4.8abc	26.6 ± 1.3c	16.6 ± 3.3abc	63.4 ± 5.2abc
Rh+15BSFFF	11.0 ± 0.8de	7.4 ± 1.7def	4.6 ± 0.5abc	17.2 ± 1.8def	9.1 ± 1.2cdef	39.5 ± 7.2abc
Rh+30BSFFF	26.3 ± 2.0ab	17.0 ± 0.8ab	10.3 ± 0.3abc	34.9 ± 1.2ab	18.9 ± 0.8a	62.9 ± 6.8abc
Rh+45BSFFF	13.2 ± 1.0cde	9.4 ± 1.4bcdef	10.0 ± 4.4abc	17.9 ± 1.8de	13.3± 0.8abcde	61.3 ± 7.6abc
Rh+15PHY	12.9 ± 2.5cde	8.8 ± 0.3cdef	2.7 ± 0.6bc	17.5 ± 1.7def	10.4 ± 1.6bcdef	35.0 ± 3.9abc
Rh+30PHY	25.8 ± 0.7ab	16.8 ± 1.8ab	18.8 ± 4.6ab	34.8 ± 0.9ab	18.1 ± 1.3a	68.1 ± 9.1abc
Rh+45PHY	21.0 ± 2.6bc	14.3 ± 2.1abcd	5.3 ± 1.4abc	26.3 ± 0.9c	16.5 ± 1.6abc	46.2 ± 2.0abc
Rh+NPK	15.4 ± 2.0cde	12.4 ± 0.7abcde	8.5 ± 2.4abc	21.1 ± 1.0cd	14.4 ± 1.0abcd	43.2 ± 3.0abc
*Df* *F/*χ2 *value* *p value*	*14* *16.87* *< 0.001*	*14* *8.87* *< 0.001*	*14* 68.72^*^ *< 0.001*	*14* *31.61* *< 0.001*	*14* *10.21* *< 0.001*	*14* 47.86^*^ *< 0.001*

15BSFFF, 30 BSFFF, 45 BSFFF = application rates equivalent to 15, 30, and 45 kg N ha^−1^ of black soldier fly frass fertilizer; 15PHY, 30PHY, 45PHY = application rates equivalent to 15, 30, and 45 kg N ha^−1^ of Phymyx commercial organic fertilizer; NPK = Mineral fertilizer (Urea, DAP, MOP); Rh = rhizobia inoculant; Control = unfertilized plot, ^*^= χ2 value. Per column, means (± standard error) followed by the same letter(s) are not significantly different at p ≤ 0.05. Df = Degrees of freedom, F value = Fisher’s value, p = probability value

The number of effective root nodules also varied significantly due to different fertilizer treatments (short rain season: F=8.87, df = 14, p< 0.001, long rain season: F=10.21, df = 14, p< 0.001) (**Table 7**). Plots amended with sole BSFFF at a rate of 30 kg N ha^-1^ produced beans with the highest number of effective root nodules (short rain season: 17 nodules, long rain season: 19 nodules), which were 13 – 14-fold, 84, 95 – 161% and 85 – 218% (p< 0.001) higher compared to those grown using the control treatment, BSFFF at a rate of 30 kg N ha^-1^ in combination with rhizobia, Phymyx at a rate of 15 kg N ha^-1^ and BSFFF applied at a rate of 15 kg N ha^-1^ either singly or in combination with rhizobia, respectively.

#### 3.3.2 Amount of nitrogen fixed

Across the two seasons, the different fertilizer treatments significantly influenced the amount of N fixed by bush beans (short rain season: χ2 = 68.72, df = 14, p< 0.001, long rain season: χ2 = 38.65, df = 14, p< 0.001) (**Table 7**). The highest quantity of N fixed by bush beans was achieved in beans grown using sole BSFFF at a rate of 30 kg N ha^-1^, which was 265% (p< 0.001) higher than N fixed in the control plots across the two seasons. For plots treated with sole BSFFF, significant differences in the quantity of N fixed were attained at rates above 15 kg ha^−1^. For Phymyx, significant (p< 0.001) differences were only achieved when the rate of 30 kg ha^−1^ was combined with rhizobia inoculant during the short rain season. Across treatments, the quantity of N fixed was higher during the long rain season compared to the values achieved during short rain season.

### 3.4 Influence of black soldier fly frass fertilizer and commercial fertilizers on bush bean reproduction and yield

#### 3.4.1 Number of flowers and pods per plant

**Figure 3:**
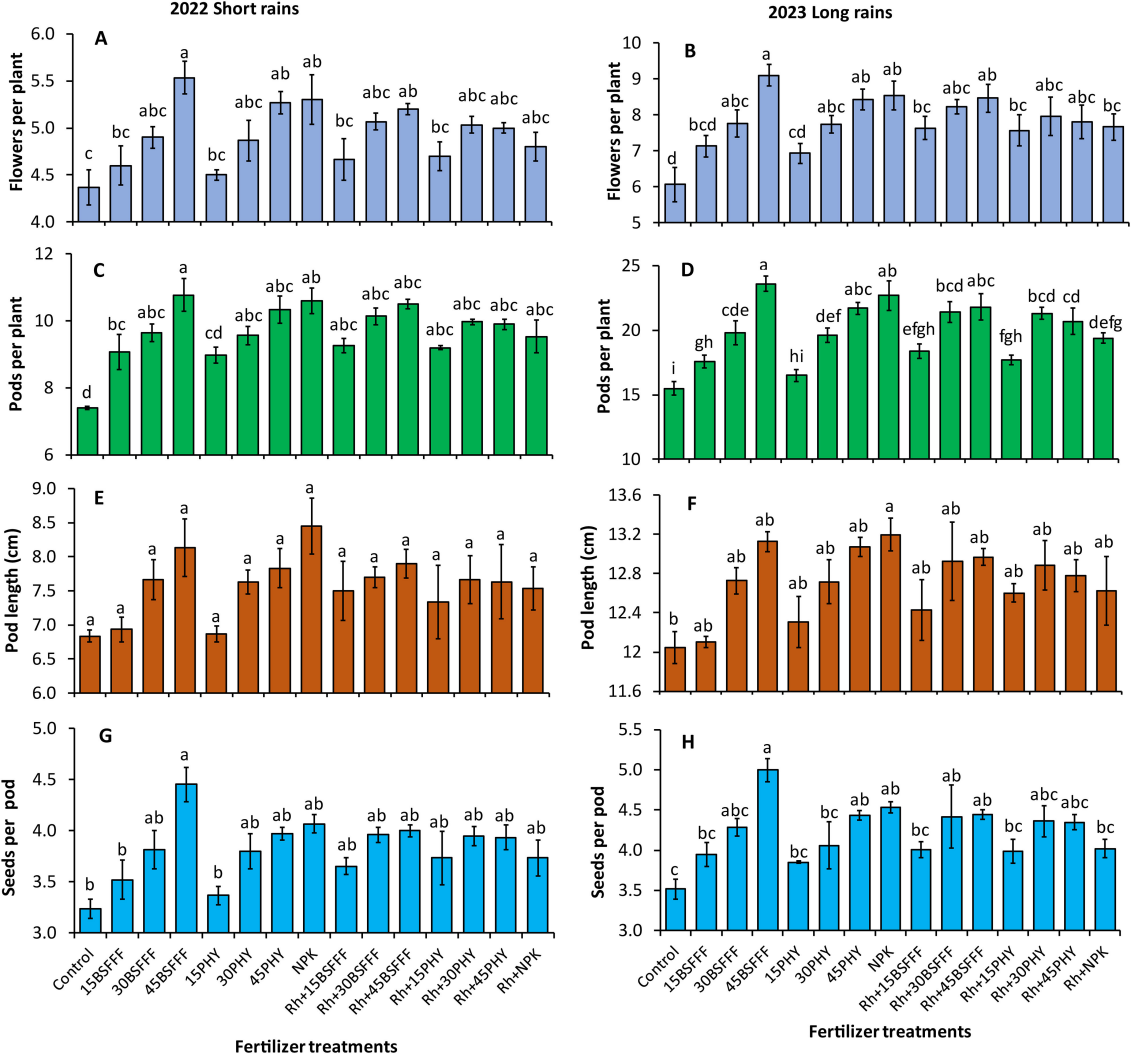
Number of flowers **(A, B)**, number of pods **(C, D)**, pod length **(E, F)**, and number of seeds per pod **(G, H)** of bush beans as influenced by different fertilizer treatments during the short rains and long rains seasons. 15BSFFF, 30 BSFFF, 45 BSFFF = application rates equivalent to 15, 30, and 45 kg N ha^−1^ of black soldier fly frass fertilizer; 15PHY, 30PHY, 45PHY = application rates equivalent to 15, 30, and 45 kg N ha^−1^ of Phymyx commercial organic fertilizer; NPK = Mineral fertilizer (Urea, DAP, MOP); Rh = rhizobia inoculant; Control = unfertilized plot. Per panel, means (± standard error) followed by the same letter(s) are not significantly different at *p* ≤ 0.05.

The different fertilizer treatments caused significant differences in the number of flowers during the study (short rain season: F = 4.22, df = 14, p< 0.001, long rain season: F = 7.30, df = 14, p< 0.001) (**Figures 3A, B**). Beans grown in soil amended with either BSFFF or Phymyx applied at sole rates of 30 and 45 kg N ha^−1^ significantly (p< 0.001) recorded higher number of flowers by 27 – 50% (BSFFF) and 20 – 39% (Phymyx) than the crops grown in unamended soil. The highest number of flowers was recorded in plots treated with sole BSFFF at a rate of 45 kg N ha^−1^, which was significantly (p< 0.001) higher than equivalent rates of 15 kg N ha^−1^ of sole Phymyx (28%) and BSFFF (32%), and combined application of rhizobia inoculant and 15 kg N ha^−1^ of either BSFFF (20%) or Phymyx (21%).

The number of bush bean pods was significantly influenced by different fertilizer treatments during the study (short rain season: F = 7.50, df = 14, p< 0.001, long rain season: F = 36.02, df = 14, p< 0.001) (**Figures 3C, D**). Bush beans grown in soil amended with sole BSFFF treatments achieved a 14–52% (p< 0.001) higher number of pods compared with beans grown in the control plots (**Figures 3C, D**). For Phymyx, only amendment at sole application rates of 30 and 45 kg N ha^−1^ produced beans with a significantly (p< 0.001) higher number of pods compared to the control plots during the short rain season. The highest number of pods was produced by beans grown in plots treated with sole BSFFF at a rate of 45 kg N ha^−1^, and this was higher (p< 0.001) than the values achieved using a combination of rhizobia inoculant with all Phymyx treatments (11 – 33%), NPK (22%), BSFFF rate of 15 N ha^−1^ (28%) and BSFFF rate of 30 kg N ha^−1^ (10%) (p< 0.001).

#### 3.4.2 Pod length and number of seeds per pod

The different fertilizer treatments significantly affected bean pod lengths during the long rain season only (short rain season: F=1.81, df = 14, P= 0.085, long rain season: F=2.71, df = 14, p = 0.0107) (**Figures 3E, F**). A significant (p< 0.001) increase in the pod sizes (10%) was realized in plots treated with sole NPK compared with the control.

The number of seeds per pod also varied significantly during the study (short rain season: F=2.84, df = 14, p = 0.008, long rain season: F=4.53, df = 14, p< 0.001) (**Figures 3G, H**). Beans grown in plots treated with sole BSFFF applied at a rate of 45 kg N ha^−1^ produced significantly (p< 0.001) higher number of seeds per pod compared to those grown in unamended soil (38 – 42%), plots amended with a combination of NPK and rhizobia inoculant (24%) during the long rain season, sole Phymyx or BSFFF applied at 15 kg N ha^−1^ during the short rain season, and Phymyx or BSFFF at a rate of 15 kg N ha^−1^ applied in combination with rhizobia inoculant during the long rain season.

#### 3.4.3 One hundred seeds weight

**Figure 4:**
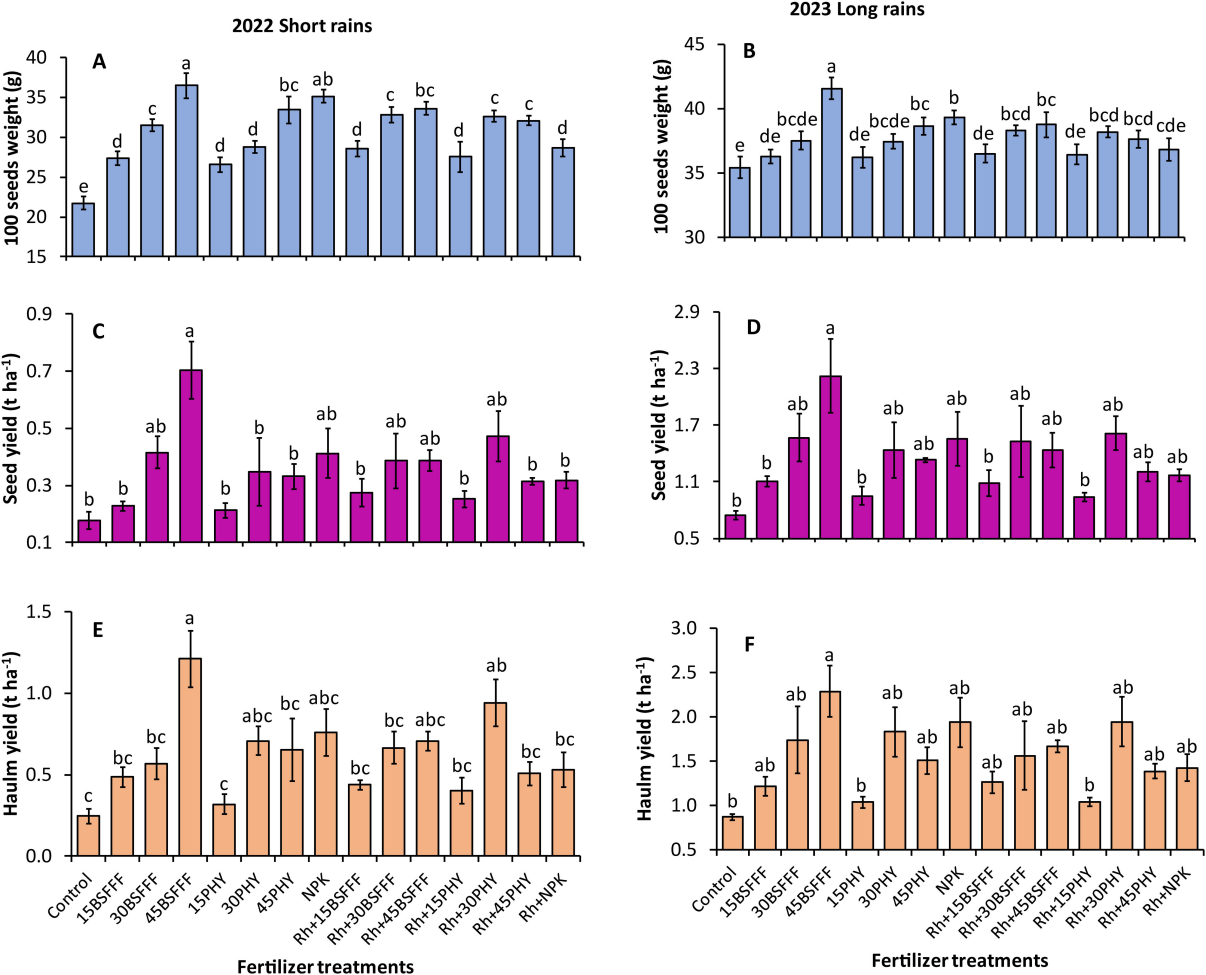
100 seed weight **(A, B)**, seed yield **(C, D)**, and haulm yield **(E, F)** of bush beans as influenced by different fertilizer treatments during the short rains and long rains seasons. 15BSFFF, 30 BSFFF, 45 BSFFF = application rates equivalent to 15, 30, and 45 kg N ha^−1^ of black soldier fly frass fertilizer; 15PHY, 30PHY, 45PHY = application rates equivalent to 15, 30, and 45 kg N ha^−1^ of Phymyx commercial organic fertilizer; NPK = Mineral fertilizer (Urea, DAP, MOP); Rh = rhizobia inoculant; Control = unfertilized plot. Per panel, mean (± standard error) followed by the same letter(s) are not significantly different at *p* ≤ 0.05.

There was a significant influence of the fertilizer treatments on the 100-seed weight of bush beans during the short rain season (F=80.10, df = 14, p< 0.001) (**Figure 4A**) and long rain season (F=14.13, df = 14, p< 0.001) (**Figure 4B**). The heaviest seeds were recorded in plots amended with sole BSFFF at a rate of 45 kg N ha^−1^, which were 68 and 18% significantly (p< 0.001) higher than the control during the short and long rain seasons, respectively. Also, Phymyx applied at 45 kg N ha^−1^ significantly (p< 0.001) increased 100-seed weight over the control plots.

The 100 seed weight achieved using sole BSFFF applied at 45 kg N ha^−1^ was significantly (p< 0.001) higher than the value achieved using an equivalent rate of Phymyx during the short rain season (9%), BSFFF at a rate of 45 kg N ha^−1^ combined with rhizobia inoculant (7 – 9%) and sole NPK (6%). It was noted that the combination of rhizobia inoculant with Phymyx at a rate of 30 kg N ha^−1^ significantly enhanced the 100 seed weight compared to the equivalent sole rate of Phymyx during the short rain season. There was a 9% (short rain season) and 7% (short rain season) (p< 0.001) increase in 100 seed weight in plots treated with a BSFFF rate of 45 kg N ha^−1^ compared with plots amended with rhizobia inoculant. Also, the application of NPK resulted in 22 and 7% significant (p< 0.001) increase in the 100 seed weight, compared with plots of rhizobia inoculant during the short and long rain seasons, respectively. Additionally, plots amended with rhizobia inoculant (p< 0.001) outperformed those treated with a Phymyx rate of 30 kg N ha^−1^ by 13% during the short rain season.

#### 3.4.4 Seed and haulm yield

There were significant differences in the seed yield of bush beans grown using different fertilizer treatments in the study (short rain season: χ2 = 58.45, df = 14, p< 0.001, long rain season: χ2 = 43.58, df = 14, p< 0.001) (**Figures 4C, D**). Application of sole BSFFF at a rate of 45 kg N ha^-1^ produced the highest bush bean yield during the short rain season (0.7 t ha^-1^) and long rain season (2.2 t ha^-1^), which were significantly (p< 0.001) higher than the values achieved using unfertilized soil by 199 – 250%. During the short rain season, the sole application of BSFFF at a rate of 45 kg N ha^−1^ produced higher (p< 0.001) bean seed yield compared to the equivalent rate of Phymyx (113%), a combination of BSFFF applied at 45 kg N ha^-1^ and rhizobia inoculant and NPK and rhizobia inoculant (123%) during the short rain season. Also, the bush bean seed yield achieved using sole BSFFF at a rate of 45 kg N ha^-1^ was significantly (p< 0.001) higher than the yields achieved using Phymyx (157 – 231%) and BSFFF (101 – 136%) applied at rates of 15 kg N ha^-1^ alone or in combination with rhizobia inoculant. The application of the BSFFF rate of 45 kg N ha^-1^ caused a 102% significant increase in seed yield compared with plots treated with rhizobia inoculant during the short rain season.

The different fertilizer treatments also caused significant differences in the haulm yield of bush beans (short rain season: χ2 = 77.48, df = 14, p< 0.001 (**Figure 4E**), long rain season: χ2 = 46.58, df = 14, p< 0.001) (**Figure 4F**). The haulm yield achieved using sole BSFFF applied at a rate of 45 kg N ha^−1^ was significantly higher than the yields obtained in the control plot (163 – 389%), equivalent rate of Phymyx in sole or combination with rhizobia (113 – 139%), rhizobia inoculant (82%) and NPK combined with rhizobia inoculant (129%) during the short rain season. Likewise, the haulm yield achieved using sole BSFFF at a rate of 45 kg N ha^−1^ was 120 – 121% higher than the values obtained using Phymyx at a rate of 15 kg N ha^−1^ either applied singly or in combination with rhizobia inoculant during the long rain season. For Phymyx, a significant (p< 0.001) increase in haulm yield relative to the control was only attained when the fertilizer was applied at 30 kg N ha^−1^ in combination with rhizobia inoculant during the short rain season. There was an 82% (p< 0.001) increase in haulm yield in plots treated with 45 kg N ha^−1^ of BSFFF compared with plots amended with rhizobia inoculant during the short rain season.

### 3.5 Multivariate analysis of relationships between bean growth and yield parameters as influenced by different fertilizer treatments

**Figure 5:**
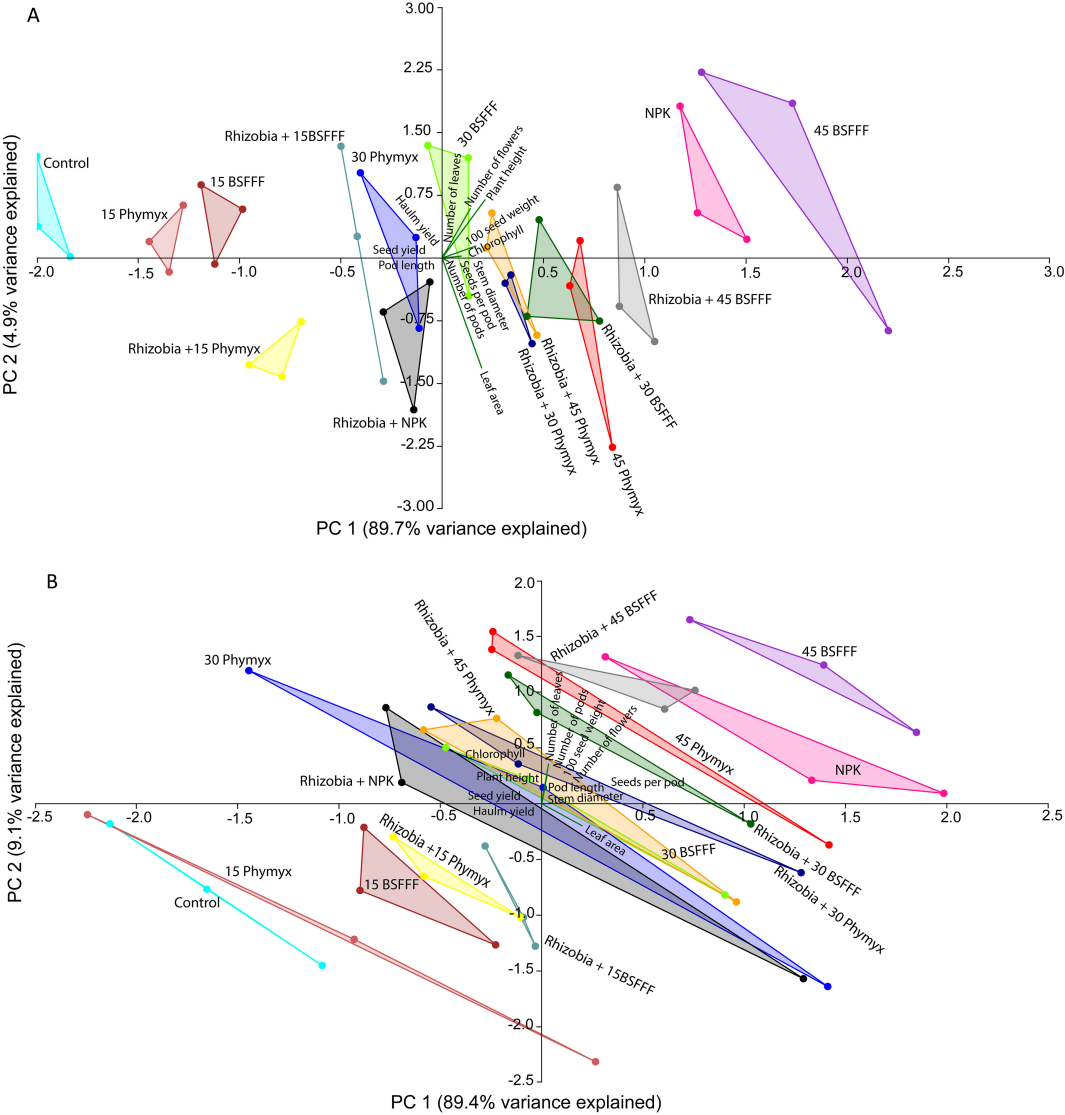
Biplots showing the relationship between bush bean growth and yield parameters for the first two principal components (PC1 and PC2) as influenced by different fertilizer treatments during short rain **(A)** and long rain **(B)** cropping seasons. 15BSFFF, 30 BSFFF, 45 BSFFF = application rates equivalent to 15, 30, and 45 kg N ha^−1^ of black soldier fly frass fertilizer; 15PHY, 30PHY, 45PHY = application rates equivalent to 15, 30, and 45 kg N ha^−1^ of Phymyx commercial organic fertilizer; NPK = Mineral fertilizer (Urea, DAP, MOP); Rh = rhizobia inoculant; Control = unfertilized plot.

The principal component analysis (PCA) revealed that the different fertilizer treatments significantly influenced bush bean growth and yield parameters (**Figure 5**). During the short rain season (**Figure 5A**), the first two components of the explained for 94.6% of the total variance. The first principal component (PC1) accounted for 89.7% while PC2 accounted for 4.9% of the total variance. The number of bean seeds per pod, number of pods, leaf area, and stem diameter positively correlated with both seed and haulm yields. During the long rain season (**Figure 5B**), the first two components represented 98.5% of the total variance. In this case, PC1 explained 89.4%, whereas PC2 explained 9.1%. Bean leaf area, plant height, and chlorophyll content were positively correlated with seed yield. On the other hand, most growth parameters, such as the stem diameter, number of leaves, pod length and number of pods positively correlated with haulm yield. The number of leaves, number of flowers, pod length, and number of pods, number, 100 seed weight, chlorophyll and plant height were positively correlated during both seasons.

### 3.6 Profitability of bush bean production using various fertilizer treatments

#### 3.6.1 Net income and gross margin

The long rain season was more rewarding economically than the short rain season (**Table 8**). Plots amended with sole NPK generated the highest net income during the long rain season, which was more than its combined application with rhizobia inoculant. Without inoculation, bush beans grown using BSFFF at rates of 15, 30, and 45 kg N ha^−1^ generated 73 – 239% higher net income compared with equivalent rates of Phymyx. With inoculation, plots treated with BSFFF generated 141– 817% more net income only at application rates of 15 and 45 kg N ha^−1^ compared with equivalent rates of Phymyx, however at a rate of 30 kg N ha^−1^ Phymyx recorded 6% more values than the equivalent rate of BSFFF. Under inoculation, BSFFF at a rate of 45 kg N ha^−1^ yielded more net income (124%) than its combined application with rhizobia inoculant. Phymyx applied at a rate of 30 kg N ha^−1^ with rhizobia inoculant recorded a relatively higher net income (13%) than without inoculation. Additionally, BSFFF at a rate of 45 kg N ha^−1^ applied alone resulted in 32% more net income than when NPK was applied with rhizobia inoculant.

**Table 8:** Economic indices (net income, gross margin, benefit cost ratio and return on investment) of bush beans production as influenced by different fertilizer treatments.

Season	Treatment	Net Income (US$ ha^−1^)	Gross Margin (%)	Benefit cost ratio (US$ ha^−1^)	Return on investment (%)
Short rains (October 2022 – January 2023)	Control	-505.6 ± 44.9ab	-170.1 ± 13.7abcd	-0.6 ± 0.05abc	-60.6 ± 5.4abc
	15BSFFF	-906.1 ± 6.8abcd	-218.3 ± 3.1abcd	-0.7 ± 0.01bc	-68.3 ± 0.5bc
	30BSFFF	-1053.7 ± 11.9bcde	-145.5 ± 4.2dabcd	-0.6 ± 0.01abc	-57.8 ± 0.7abc
	45BSFFF	-1013.1 ± 29.8bcde	-88.9 ± 6.7ab	-0.4 ± 0.01ab	-43.8 ± 1.3ab
	15PHY	-997.6 ± 18.1def	-301.9 ± 9.4abcd	-0.7 ± 0.01bc	-74.4 ± 1.2bc
	30PHY	-1145.8 ± 11.9ef	-248.4 ± 5.1abcd	-0.7 ± 0.01bc	-68.1 ± 0.5bc
	45PHY	-2327.1 ± 13.7g	-398.1 ± 7.0cd	-0.8 ± 0.00c	-79.2 ± 0.5bc
	NPK	-339.0 ± 13.7a	-57.3 ± 2.0a	-0.3 ± 0.01a	-30.7 ± 1.2a
	Rh+15BSFFF	-843.3 ± 18.1abcd	-166.3 ± 5.2abcd	-0.6 ± 0.01abc	-62.4 ± 1.3abc
	Rh+30BSFFF	-1128.2 ± 18.1cdef	-161.9 ± 5.9abcd	-0.6 ± 0.02abc	-61.2 ± 1.0abc
	Rh+45BSFFF	-1695.3 ± 44.9f	-375.8 ± 17.1bcd	-0.7 ± 0.02bc	-72.5 ± 1.9bc
	Rh+15PHY	-1095.5 ± 6.8bcdef	-235.4 ± 2.7abcd	-0.7 ± 0.00bc	-70.2 ± 0.4bc
	Rh+30PHY	-1390.2 ± 31.4def	-176.0 ± 12.6abcd	-0.6 ± 0.01abc	-61.5 ± 1.4abc
	Rh+45PHY	-2374.6 ± 18.1g	-411.5 ± 9.5d	-0.8 ± 0.01c	-80.2 ± 0.6c
	Rh+NPK	-526.6 ± 6.8abc	-91.0 ± 1.5abc	-0.5 ± 0.01abc	-47.5 ± 0.6abc
	*Df* *F/*χ2 *value* *p value*	*14* 368.32^*^ *< 0.001*	*14* 50.59^*^ *< 0.001*	*14* 63.26^*^ *< 0.001*	*14* 63.26^*^ *< 0.001*
Long rains (March 2023 – June 2023)	Control	502.9 ± 77.2a	40.8 ± 3.5ab	0.7 ± 0.1ab	70.1 ± 10.8ab
	15BSFFF	538.9 ± 33.1a	29.7 ± 1.3ab	0.4 ± 0.02b	42.3 ± 2.6b
	30BSFFF	748.8 ± 419.5a	24.7 ± 13.4ab	0.4 ± 0.2b	41.0 ± 22.9b
	45BSFFF	1268.8 ± 643.3a	30.1 ± 13.4ab	0.5 ± 0.3b	53.2 ± 26.7b
	15PHY	240.1 ± 162.4a	13.6 ± 8.3ab	0.2 ± 0.1b	18.2 ± 12.3b
	30PHY	434.1 ± 484.9a	8.7 ± 23.5ab	0.2 ± 0.3b	22.5 ± 25.1b
	45PHY	374.4 ± 207.3a	12.0 ± 5.9ab	0.1 ± 0.08b	14.8 ± 8.2b
	NPK	1601 ± 467.9a	59.8 ± 7.7a	1.7 ± 0.5a	167.5 ± 48.9a
	Rh+15BSFFF	489.6 ± 231.5a	24.6 ± 10.9ab	0.4 ± 0.2b	37.8 ± 17.9b
	Rh+30BSFFF	659.9 ± 618.2a	17.3 ± 18.7ab	0.4 ± 0.3b	35.7 ± 33.5b
	Rh+45BSFFF	566.5 ± 213.1a	18.2 ± 6.1ab	0.2 ± 0.08b	23.6 ± 8.9b
	Rh+15PHY	203.3 ± 72.7.4a	12.7 ± 4.3ab	0.2 ± 0.05b	15.1 ± 5.4b
	Rh+30PHY	702.2 ± 300.0a	24.5 ± 8.9ab	0.4 ± 0.2b	35.9 ± 15.4b
	Rh+45PHY	61.8 ± 113.8a	2.0 ± 4.4b	0.02 ± 0.04b	2.4 ± 4.4b
	Rh+NPK	965.2 ± 106.4a	49.8 ± 2.8ab	1.0 ± 0.1ab	100.3 ± 11.1ab
	*Df* *F/*χ2 *value* *p value*	*14* 19.97^*^ *0.131*	*14* *2.16* *0.038*	*14* 55.70^*^ *< 0.001*	*14* 55.70^*^ *< 0.001*

Cost of production: Phymyx = US$ 0.45 kg^−1^, BSFFF = US$ 0.29 kg^−1^, Urea = US$ 0.94 kg^−1^, DAP = US$ 0.93 kg^−1^, MOP = US$ 0.84 kg^−1^, Rhizobia inoculant = US$ 4.08 100 g^−1^, Bean seeds = US$ 2.3 kg^−1^, Labor = US$ 1.25 hour^−1^. Sources of revenue: Bean seeds = US$ 1.65 kg^−1^. **^2^** 15BSFFF, 30 BSFFF, 45 BSFFF = application rates equivalent to 15, 30 and 45 kg N ha^−1^ of black soldier fly frass fertilizer; 15PHY, 30PHY, 45PHY= application rates equivalent to 15, 30 and 45 kg N ha^−1^ of Phymyx commercial organic fertilizer; NPK = Mineral fertilizer (Urea, DAP, MOP); Rh = rhizobia inoculant; Control = unfertilized plots, ^*^= χ2 value, Df = Degrees of freedom, F value = Fisher’s value, p = probability value. Per column, means (± standard error) followed by the same letter(s) are not significantly different at p ≤ 0.05.

The highest gross margin was recorded in plots treated with sole NPK, which was 20% more than values generated from its combined application with rhizobia inoculant (**Table 8**). The gross margin achieved using sole BSFFF at a rate of 45 kg N ha^−1^ was 65% and 150% higher compared to when the same fertilizer was combined with rhizobia inoculant and the value achieved using similar rate of Phymyx, respectively. Combined application of Phymyx at 30 kg N ha^−1^ with rhizobia inoculant yielded 181% higher gross margin compared to its sole application.

#### 3.6.2 Benefit-cost ratio and return on investment

Bush beans grown using sole NPK yielded the highest benefit-cost ratio (1.7) which was 67% higher than the inoculated ones (**Table 8**). Plots amended with sole BSFFF at a rate of 45 kg N ha^−1^ generated 253% more benefit-cost ratio compared with the ones that had received an equivalent rate of Phymyx. In addition, when BSFFF at a rate of 45 kg N ha^−1^ was applied singly, the benefit-cost ratio increased by 121% more than its combined application with rhizobia inoculant.

Plots amended with sole NPK generated the highest return on investment (ROI), which was 67% more than its combined application with rhizobia inoculant. Plots treated with sole NPK yielded the highest ROI that was significantly (p< 0.001) higher than the values achieved using BSFFF (3 – 7-fold) and Phymyx (4.7 – 70-fold) treatments. The sole BSFFF treatments yielded 82 – 260% higher ROI compared to equivalent rates of Phymyx. There was a decline in ROI as the application rates of BSFFF and Phymyx increased, but the decreases were not significant (**Table 8**).

## 4 Discussion

### 4.1 Influence of black soldier fly frass fertilizer and commercial fertilizers on bush bean growth and yield

The higher growth rate and yield of bush beans grown in fertilized soil compared to the unfertilized soil indicate the significant role of fertilizers in boosting crop production in degraded soils which dominate farmlands across Africa and the tropics (Wortmann et al., 2019; Nungula et al., 2023). As demonstrated, BSFFF improved the growth of bush beans better than commercial fertilizers, which is in tandem with previous studies that have shown the benefits of insect frass fertilizers in boosting the growth of different crops (Anyega et al., 2021; Beesigamukama et al., 2020a). This could be attributed to a faster release of nutrients such as nitrogen and phosphorus for plant uptake, which are critical for physiological processes such as chlorophyll formation (Mulambula et al., 2019; Xiang et al., 2012). Past studies have reported taller plants and higher chlorophyll content in crops grown using BSFFF, compared to commercial fertilizers (Klammsteiner et al., 2020; Quilliam et al., 2020). The comparable performance of frass fertilizer and mineral fertilizer in terms of number of leaves and stem diameter has been previously reported (Klammsteiner et al., 2020) and illustrates that frass fertilizer can be a sustainable alternative to mineral fertilizers. Also, the growth hormones present in BSFFF and the ability to suppress plant pests (Anedo et al., 2024; Kisaakye et al., 2024; Tanga and Kababu, 2023) and diseases (Kemboi et al., 2022; Lagat et al., 2021) could have mostly contributed to improved growth of bush beans observed during the study.

On the other hand, the combined application of the organic fertilizers and rhizobia inoculant did not significantly affect the growth of beans compared to the sole application of BSFFF at a rate of 45 kg N ha^−1^. This could mostly be due to underlying effects such as incompatibility of the bio-fertilizer with the organic fertilizers. The rhizobia inoculant could have also faced competition from the indigenous rhizobacteria, which counteracted the effect on the growth of bush beans (Zaheer et al., 2022). Bio-fertilizer performance is also affected by dynamics in soil conditions such as temperature, salinity, pH, and moisture (Zaheer et al., 2022; Mwadalu et al., 2022; Mohammadi et al., 2012). It is further reported that bush beans can be inoculated by more than one species of rhizobia (Sousa et al., 2022; Cordeiro et al., 2017; Gicharu et al., 2013), therefore, limiting growth through negative interaction when elite strains of rhizobia were introduced. This, therefore, illustrates the inefficiency of elite strains of rhizobacteria on the growth of bush beans in addition to being host-specific, thus limiting their effectiveness in bush bean production.

Our findings revealed positive correlations between bean growth parameters and seed yield. The highest number of flowers, pods, and seeds per pod achieved by beans grown in soils amended with sole BSFFF treatment at a rate of 45 kg N ha^−1^ could be attributed to the ability of insect frass fertilizer to promote flower formation and pollination (Barragán-Fonseca et al., 2022). Additionally, the higher number of pods could be attributed to the higher chlorophyll content due to optimal release of N and P, and a resultant transfer of photosynthates for pod and seed formation (Goswami et al., 2019). The higher K and P concentrations, as well as micronutrients in BSFFF compared with commercial organic fertilizer, further explains the better flower and seed reproduction in beans grown using BSFFF compared to the commercial organic fertilizer (Phymyx) (Kakon et al., 2016; Dhanjal et al., 2003).

Bush beans grown during the long rain season accumulated more nutrients compared to their counterparts in the short rain season, due to favorable rainfall that enhanced faster release and uptake of nutrients by bush bean plants. This aligns with previous studies that demonstrated higher nutrient uptake of chickpeas under a sufficient irrigation regime than the water-stressed soils (Chtouki et al., 2022). This study revealed the benefits of using sole BSFFF for sufficient nutrient uptake, clearly evidenced by the higher macro nutrient uptake compared with those grown using commercial fertilizers. It is well documented that the novel insect frass fertilizer improves soil health in terms of pH, and nutrient availability, and even curbs the challenge of moisture stress (Beesigamukama et al., 2020b; Adin Yéton et al., 2019; Kagata and Ohgushi, 2012). Previous studies further found that the fertilizer is rich in beneficial microorganisms, higher carbon levels, and mineralization rate to enhance faster release and uptake of nutrients compared to commercial fertilizers (Agustiyani et al., 2021; Beesigamukama et al., 2020a, b; Gold et al., 2020). The higher nutrient uptake in beans grown using sole BSFFF compared with commercial fertilizers, could be due to higher BNF success and better root formation linked to P availability within the rhizosphere (Beesigamukama et al., 2021b; Xiang et al., 2012). This could have contributed to higher bush bean yield from BSFFF-amended plots compared to those fertilized using commercial fertilizers (Devi et al., 2012), and aligns with previous studies that reported increased yield of crops such as maize, tomatoes, and French beans using BSF frass fertilizer (Anyega et al., 2021; Beesigamukama et al., 2020a). Therefore, the highest bean yield associated with sole BSFFF at a rate of 45 kg N ha^−1^ implies that frass fertilizer can be relied on as a substitute for commercial fertilizers for improved bush bean yield.

Our study endorses the optimal application of sole BSFFF at a rate of 45 kg N ha^−1^ to be adopted in bush bean cropping systems to attain a yield potential of 1.5 – 2 tonnes ha^-1^ (Mukankusi et al., 2019; Kalyebara and Buruchara, 2008). Even though the combined application of rhizobia inoculant and BSFFF did not increase bush bean yield better than a sole dose of BSFFF, it was noted that the integrated application of 30 kg N ha^−1^ BSFFF with rhizobia inoculant provides the starter dose required for BNF process and boosts the crop yield. Therefore, stakeholders along the bush bean value chain can adopt either sole BSFFF at a rate of 45 kg N ha^−1^ or use a lower rate of 30 kg N ha^−1^ combined with rhizobia inoculant to reduce reliance on expensive and unsustainable commercial fertilizers. This will go a long way in boosting bean productivity and improving food and nutrition security, especially among smallholder farmers. It is crucial to note that for best results, BSFFF should be accompanied by other agronomic practices such as timely planting, plant spacing, weed control, pest, and disease control as well as timely harvesting to eliminate cases of yield loss.

### 4.2 Biological nitrogen fixation and economic returns to bush bean production using BSF frass fertilizer and commercial fertilizers

The higher number of effective root nodules and quantity of N fixed achieved by bush beans grown in soil amended using sole BSFFF compared to commercial rhizobia inoculant and organic fertilizers highlight the superiority of BSFFF in boosting BNF and improving nitrogen cycling in legume cropping systems. The better nodulation and N fixation associated with BSFFF treatment could be largely attributed to the positive role of BSFFF in creating conducive soil conditions for the BNF process. The high pH values of BSFFF could have reduced soil acidity which unlocked P making it available for uptake and availed calcium for the formation of nod factors necessary for the attachment of rhizobia to root surfaces, as previously reported by Beesigamukama et al. (2021b). Studies have found that nodules are greater sinks for P compared to shoots and roots of legumes (Mulambula et al., 2019; Shahid et al., 2009) due to the critical role of phosphorus in the formation of energy required for effective nodulation and BNF among pulse crops (Agustiyani et al., 2021). The BSFFF also supplies micronutrients such as molybdenum (Mo) and iron (Fe) (Beesigamukama et al., 2023) which are required for nodulation and BNF success (Nasar et al., 2021; Hänsch and Mendel 2009). Further, the BSFFF is rich in ammonium (NH_4_^+^) (Beesigamukama et al., 2021b), which is highly preferred by microorganisms as an energy source during the BNF process. Conversely, the lower nitrate (NO_3_^-1^) concentration in plots of sole BSFFF could have enhanced BNF more than commercial fertilizers since high NO_3_^-1^ is toxic to BNF, as it subdues the expression of nitrogenase activities (Reinprecht et al., 2020; Mingotte et al., 2019; Faghire et al., 2011).

Past studies have shown the greater populations of beneficial microbiota such as nitrifying bacteria contained in the BSFFF (Gold et al., 2020; Surendra et al., 2020; Choi and Hassanzadeh, 2019), which could have played a key role in ensuring effective nodulation and BNF, compared to commercial fertilizers. Beyond nutrients supply, the insect frass fertilizer also improves other soil properties which are key for BNF success (Beesigamukama et al., 2023; Poveda et al., 2019). Improved water-holding capacity, tolerance to moisture stress, proper soil aeration, and soil aggregation have been reported in soils amended with BSFFF and could have enhanced the activities of native and active rhizobia for improved nodulation and BNF process (Beesigamukama et al., 2020b; Pagliai et al., 2004). Studies by Abiya et al. (2022) have demonstrated the benefits of BSFFF in boosting tolerance to moisture stress, while Beesigamukama et al. (2020b) reported higher moisture storage in soils amended with BSFFF. Additionally, the BSFFF is known to suppress soil-borne pathogens that cause diseases in different crops, including beans (Kemboi et al., 2022; Lagat et al., 2021; Quilliam et al., 2020). Our sister studies have demonstrated the high potential of BSFFF in suppressing soil-dwelling pests such as nematodes root maggots (Anedo et al., 2024; Kisaakye et al., 2024; Tanga and Kababu, 2023), and an array of above ground pests (Abiya et al., 2022), thus protecting and ensuring the health of bean crops for high BNF success. Nonetheless, future studies will be necessary to validate the soil health benefits of BSFFF, survival of rhizobia in soil, carbon sequestration, and greenhouse gas mitigation in bean cropping systems.

The lower number of effective nodules and lower N fixation associated with rhizobia inoculant could be largely attributed to low populations of effective strains, incompatibility, competition from the native nitrifying bacteria, and unfavorable soil conditions in terms of micronutrient deficiency and moisture stress (Divito and Sadras, 2014; Hungria et al., 2013; Ndakidemi et al., 2006). On the other hand, the lower BNF associated with commercial organic fertilizer treatments could be due to lower populations of rhizobia and limited nutrient supply (Fernández-Calviño et al., 2010; Widmer et al., 2006). The findings reveal the ineffectiveness of rhizobia inoculant for BNF in degraded soils and bean cropping systems ravaged by climate change impacts. The higher BNF success and bean yield achieved using BSFFF imply that to ensure high BNF success in highly weathered soils, it is better to create favorable soil conditions and stimulate activities of native rhizobia populations, rather than supplying elite rhizobia strains in degraded soils with adverse environmental conditions.

Our findings show that BSFFF applied at the rate of 30 kg N ha^−1^ could boost the activities of indigenous nitrogen-fixing bacteria and fix appreciable amounts of nitrogen for individual use by legumes, while sparing some for other crops in rotation. The highest quantity of nitrogen fixed (82.9 kg N ha^−1^) attained through the use of sole BSFFF at a rate of 30 kg N ha^-1^, was higher than the recommended N rate of 40 kg N ha^−1^ for bush bean production (Chekanai et al., 2018), indicating that BSFFF can be relied on to boost the supply of the nitrogen required for the entire growth cycle of beans.

The higher economic returns achieved using NPK fertilizer compared to organic fertilizers (Phymyx and BSFFF) and biofertilizers have been previously reported and could be attributed to higher labor costs associated with organic fertilizer production and application (Beesigamukama et al., 2021c; Mucheru-Muna et al., 2014). Because the BSFFF is an emerging input, its price is slightly high due to high demand; it is anticipated that scaling production of BSFFF will reduce the price and increase the profits of crop production. On the other hand, previous economic assessments (Beesigamukama et al., 2021c) have revealed that a circular economy model involving the direct use of locally produced BSF frass fertilizer for crop production boosts profit margins compared to NPK, which is critical especially for smallholder farmers that are often time-rich but financially constrained. Furthermore, it is anticipated the residual benefits of BSFFF on soil health will reduce the need for seasonal application, leading to higher profit margins. On the other hand, the higher net income and profit margins associated with BSFFF compared to Phymyx could be attributed to its superior nutrient quality, and lower cost compared with Phymyx. Similar findings were reported by Tanga et al. (2022) where the BSFFF yielded higher economic returns to maize production compared to commercial organic fertilizers. This illustrates the potential of substituting the costly yet less effective commercial organic fertilizers (Gram et al., 2020).

Our findings have outlined the critical role played by BSFFF as a sustainable organic input with reduced ecological footprint compared to commercial fertilizers. Compared to mineral fertilizers, insect frass fertilizer offers multiple benefits and plays a critical role in rejuvenating the highly weathered and degraded soils of SSA and other tropical regions. This novel and climate-smart fertilizer technology is holistic in nature as a circular economy strategy for recycling organic waste residues into high-quality fertilizer for crop production and insect-based livestock feed while boosting household income, conserving the environment, and creating jobs and opportunities for women, youth, and other marginalized communities. Ultimately, the insect-driven fertilizer technology contributes to food and nutrition security, poverty reduction, responsible consumption, biodiversity conservation, climate change mitigation, and the realization of several sustainable development goals in general.

## 5 Conclusion

Our study provides, for the first time, evidence of the efficacy of black soldier fly frass fertilizer in enhancing bush bean growth, yield, and biological N fixation. The highest bush bean growth rate, nutrient uptake, and yield were achieved from soil amended with BSF frass fertilizer applied at a rate equivalent to 45 kg N ha^-1^ while the highest net income was achieved at a rate equivalent to 15 kg N ha^-1^. We have demonstrated that BSF frass fertilizer application at rates equivalent to 30 kg N ha^-1^ can boost the biological N fixation process and enable bush beans to fix enough nitrogen required for optimal growth and yield, thus eliminating the necessity for inorganic fertilizer or biofertilizer application in bean cropping systems. Our findings show that boosting biological nitrogen fixation in bush beans requires strategies that restore soil health to stimulate indigenous rhizobia populations, rather than supplying elite strains of rhizobia in soils with multiple degradation challenges. Therefore, the adoption of BSF frass fertilizer in bush bean cropping systems will counteract the high costs of mineral fertilizers, contribute to sustainable soil health management, and accelerate the transition to circular, regenerative, and climate-smart agri-food systems. Nevertheless, further studies are warranted to determine nodule occupancy of bush beans grown in soils amended using BSF frass fertilizer, and the mid-long-term effects of this fertilizer on soil health, carbon sequestration, and greenhouse gas emissions.

## Data Availability

The original contributions presented in the study are included in the article/supplementary material. Further inquiries can be directed to the corresponding authors.
